# Application and Evaluation of an Expert Judgment Elicitation Procedure for Correlations

**DOI:** 10.3389/fpsyg.2017.00090

**Published:** 2017-01-31

**Authors:** Mariëlle Zondervan-Zwijnenburg, Wenneke van de Schoot-Hubeek, Kimberley Lek, Herbert Hoijtink, Rens van de Schoot

**Affiliations:** ^1^Department of Methods and Statistics, Utrecht UniversityUtrecht, Netherlands; ^2^Schreuder College Location Villeneuvestraat, Horizon Jeugdzorg en Onderwijs [Horizon Youth Care and Education]Rotterdam, Netherlands; ^3^CITO Institute for Educational MeasurementArnhem, Netherlands; ^4^Optentia Research Focus Area, North-West UniversityPotchefstroom, South-Africa

**Keywords:** expert judgment, elicitation procedure, correlation, informative priors, Bayesian analysis

## Abstract

The purpose of the current study was to apply and evaluate a procedure to elicit expert judgments about correlations, and to update this information with empirical data. The result is a face-to-face group elicitation procedure with as its central element a trial roulette question that elicits experts' judgments expressed as distributions. During the elicitation procedure, a concordance probability question was used to provide feedback to the experts on their judgments. We evaluated the elicitation procedure in terms of validity and reliability by means of an application with a small sample of experts. Validity means that the elicited distributions accurately represent the experts' judgments. Reliability concerns the consistency of the elicited judgments over time. Four behavioral scientists provided their judgments with respect to the correlation between cognitive potential and academic performance for two separate populations enrolled at a specific school in the Netherlands that provides special education to youth with severe behavioral problems: youth with autism spectrum disorder (ASD), and youth with diagnoses other than ASD. Measures of face-validity, feasibility, convergent validity, coherence, and intra-rater reliability showed promising results. Furthermore, the current study illustrates the use of the elicitation procedure and elicited distributions in a social science application. The elicited distributions were used as a prior for the correlation, and updated with data for both populations collected at the school of interest. The current study shows that the newly developed elicitation procedure combining the trial roulette method with the elicitation of correlations is a promising tool, and that the results of the procedure are useful as prior information in a Bayesian analysis.

## 1. Introduction

“Expert judgement has always played a large role in science and engineering. Increasingly, expert judgment is recognized as just another type of scientific data …” (Goossens et al., 2008, p. 236).

This quote is the result of 15 years of developing and applying expert judgment elicitation procedures at TU Delft in the Netherlands. In the sectors of, for example, nuclear applications, chemical industries, water pollution, volcano eruptions, space shuttles, aviation, health, banking, and occupational hazards over 800 experts have conducted elicitations on over 4000 variables (Goossens et al., 2008). In social science, however, expert judgment is seldom used for estimation and inference, especially not in combination with data (see Spiegelhalter et al., 2000; O'Hagan et al., 2006 for a few examples in health care). This may be partly explained by the fact that the Bayesian framework that allows for the inclusion of prior knowledge elicited from experts in data analyses was adopted much earlier and on a far greater scale by fields of science, technology, engineering, and mathematics as compared to social science, arts, and humanities (Van de Schoot et al., 2016). Nevertheless, the use of Bayesian statistics is increasing in social science as well.

**Figure 1:**
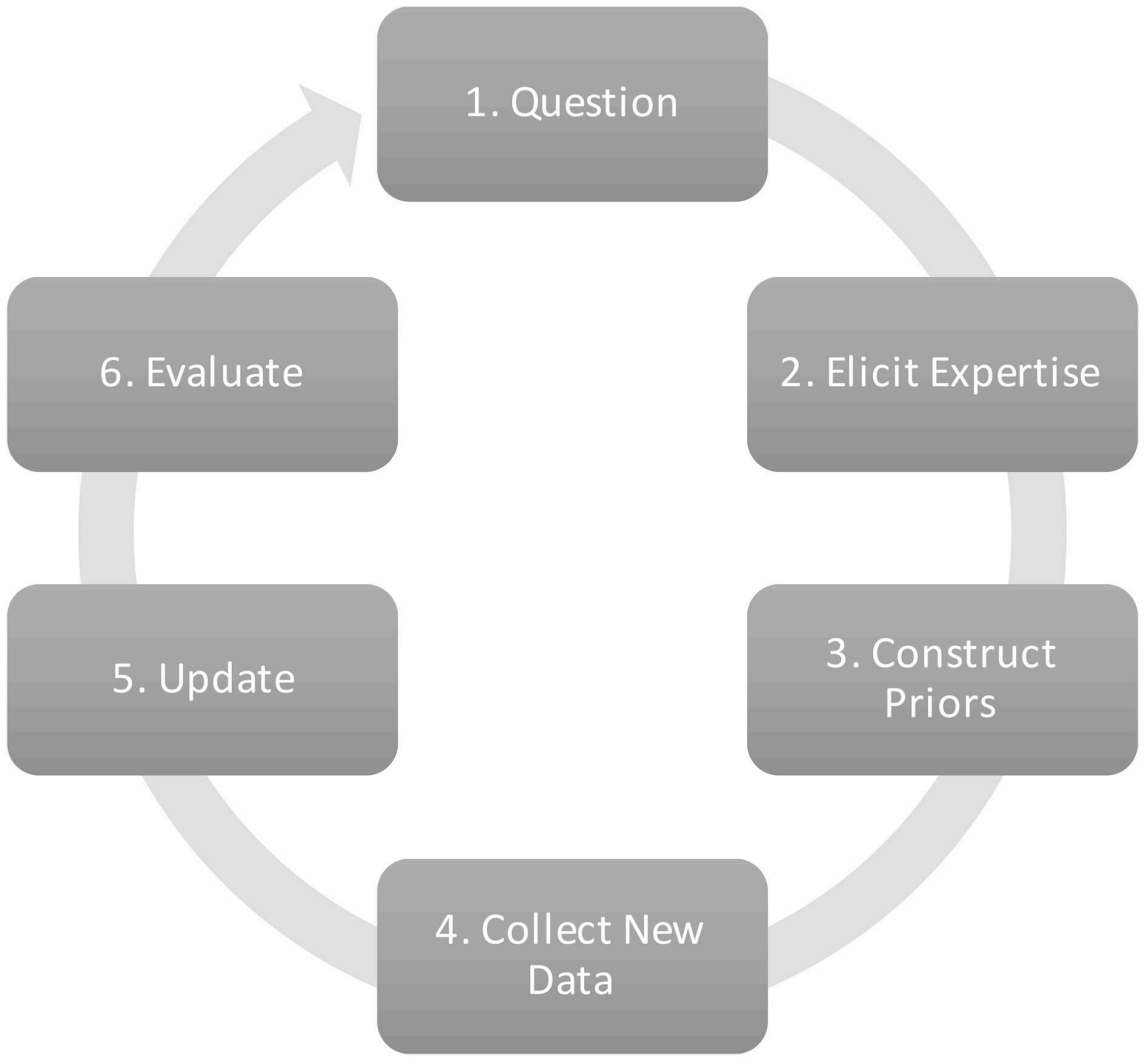
**Research cycle to update expert judgments with new data**.

In Bayesian statistics, a prior distribution containing probable values for each parameter of a model is updated with data, resulting in a posterior distribution: an updated summary of the knowledge about the model parameters^1^. Expert judgments can be a useful source of prior information, especially when data is scarce (Hampson et al., 2014). Small samples contain a limited amount of information, and the reliability of the data may be questionable. Expert judgments can complement the information from the data. Additionally, updating current expert judgments with new data can also be a research goal in itself. The updated result can increase confidence in original views of experts, or adapt these views. In the current study, we focus on the elicitation of a correlation between two variables. The correlation–our key parameter –is modeled in a bivariate normal distribution that consist of two means, and two standard deviations next to the correlation parameter itself. Figure 1 shows the research cycle that can be followed when expert judgments for a key parameter are to be updated with data.

When the research objective is to update expert judgments with current data, these judgments have to be elicited first (see Figure 1, step 2). The elicitation of judgments is a sensitive process, because the human mind tends to employ easy-to-use strategies that are not necessarily rational or optimal (Van Lenthe, 1993; O'Hagan et al., 2006). The elicitation of correlations between variables has received considerable attention in fields other than social science. Kraan (2002) and O'Hagan et al. (2006) describe, for example, (1) a method where stregth of the relationship between variables is expressed on a 7-point Likert scale, (2) a method where the expert is requested to provide Spearmans's correlation, (3) a method where the probability of concordance is assessed (further explained in a later section), and (4) a method that requests conditional quantile estimates. Clemen et al. (2000) evaluated six methods to elicit judgments about correlations with respect to accuracy, variation among experts, and difficulty. The best method according to their study was to simply ask experts to report a correlation. However, many others are critical to the capability of the human mind to assess a correlation (Gokhale and Press, 1982; Morgan et al., 1992; O'Hagan et al., 2006). It is clear that determining a correlation is not an easy task. Hence, instead of eliciting a point estimate as in the above methods, we consider it important to elicit a full distribution that captures the experts' uncertainty as well.

One way to elicit continous distributions is to ask the expert to specify fractiles or quantiles of the distribution of interest such as the 5, 50, and 95th. After a training with respect to quantiles, a question to obtain the 5th percentile for the mean of IQ in a specific population may be: “Can you determine a value such that the mean of IQ is 5% likely to be less than this point and 95% likely to be greater than this point?” (O'Hagan et al., 2006). Such a question should be asked for all desired quantiles. Alternatively, one could ask for multiple quantiles at once, for example: “To capture your uncertainty please provide the 5, 25, 50, 75, and 95th percentiles of your uncertainty distribution” (Morales-Nápoles, 2010, p. 82). Morales-Nápoles (2010) used this method to elicit a distribution for a correlation. After the elicitation phase, distributions are fitted to the elicited quantiles (Cooke and Goossens, 1999).

**Figure 2:**
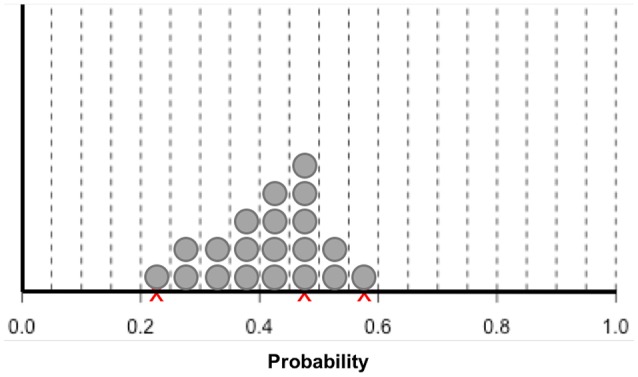
**Bins and chips method according to Johnson et al. (2010b)**. Experts are first asked to indicate their estimation of survival probability with an X. Subsequently, the experts are asked to indicate the lower and upper limits of their estimate using an X. Finally, experts are given 20 stickers, each representing 5% probability. Experts are asked to place the stickers in the intervals to indicate the weight of belief for their survival estimates.

Another way to obtain uncertainty distributions is the trial roulette method (Gore, 1987). Experts are provided with a number of “chips” to allocate probability to bins of a histogram (see Figure 2). With 20 chips, each chip represents five percent probability. The number of chips placed over a certain value reflects how probable the value is according to the expert. Several variants on this method have been developed and evaluated. It appears that the trial roulette response format improves accuracy and counters overconfidence (Goldstein et al., 2008; Haran and Moore, 2010, 2014; Goldstein and Rothschild, 2014). Johnson et al. (2010b) evaluated the trial roulette method by eliciting judgments from academic specialists about probabilities of 3-year survival with and without medicine for pulmonary hypertension patients, and concluded that the trial roulette method is feasible, has face validity, is internally valid, and has good intrarater reliability. Compared to the quantile method, the trial roulette method provides immediate visual feedback to experts, which can reduce bias, and improve reliability and validity (Clemen et al., 2000; Haran and Moore, 2014).

The current study is the first to combine the trial roulette method to elicit distributions with insights from the literature on eliciting correlations. We will follow Johnson et al. (2010b) in an effort to evaluate our elicitation procedure. Moreover, the current study illustrates the application of the procedure, and the use of the elicited distributions in a social science application.

## 2. Evaluation of the elicitation procedure

In the current section we evaluate the elicitation procedure using the responses and feedback from experts who participated in an illustrative elicitation event according to the elicitation procedure. The elicitation concerned the correlation between cognitive potential (i.e., IQ) and educational performance at a specific school in the Netheralnds that provides special education to youth who show severe behavioral problems. This school serves two important populations: youth with an autism spectrum disorder (ASD), and youth with diagnoses other than ASD from the diagnostic and statistical manual of mental disorders (American Psychiatric Association, 1994). Educational performance was operationalized as the youth's didactic age equivalent divided by didactic age (DAE/DA). This measure is widely used among behavioral scientists working in Dutch education to assess academic progress relative to received months of education.

### 2.1. Material and methods

#### 2.1.1. Participants

In our illustration, the expert identification and selection were conducted at once by our key informant regarding the subject matter: WH. WH is a school psychologist who works with the population of interest, and is a member of the Dutch Association of Psychologists—section Crisis Response Network School Psychologists. WH selected six behavioral scientists working on schools for youth with severe behavioral problems in The Netherlands, who were familiar with the school and population of interest. Following Hora and Von Winterfeldt (1997), the selection was based on expertise, understanding of the problem area, and statistical understanding. All six experts were contacted by e-mail, and agreed to participate, but two of them could not attend the scheduled meeting. The attending experts were 27, 33, 40, and 46 years old females, and were working as behavioral scientists for 4, 9, 18, and 16 years, respectively.

#### 2.1.2. Expert judgment elicitation

The procedure to elicit judgments about correlations is a semi-structured face-to-face group interview. The semi-structured setup of the procedure implies that experts are actively invited to contribute. Furthermore, the facilitator responds to questions and elaborates explanations such that everything is clear to each of the experts, which promotes validity. Group interviews additionally improve judgment synthesis through the interaction that occurs among experts, and may diminish overconfidence (O'Hagan et al., 2006; Johnson et al., 2010a).

The procedure was developed through repeated communication with colleagues at the department of methods and statistics at Utrecht University (UU), students of the research masters methodology and statistics for the behavioral, biomedical, and social sciences, and our key informant WH. Furthermore, a pilot test was conducted with students of the UU research masters Development and Socialization in Childhood and Adolescence. Details on the development of the procedure are provided as online Supplementary Material (Part I). Based on O'Hagan et al. (2006), Johnson et al. (2010a), and Johnson et al. (2010b), the elicitation procedure consists of seven phases: (1) motivation, (2) clarification, (3) education, (4) instruction, (5) background questions, (6) elicitation of expert judgments, and (7) evaluation. Instructions for the elicitation procedure are provided in Appendix 1. The material supporting the elicitation procedure is provided as online Supplementary Material (Part II).

The first four phases of the elicitation procedure serve to improve experts' motivation for the elicitation task, and to improve their understanding of the elicitation subject, correlations, and the elicitation procedure. These elements have been shown to improve validity of elicitation processes (Clemen et al., 2000; O'Hagan et al., 2006; Johnson et al., 2010a). Experts are asked for their knowledge on the topics of interest, and are invited to complement each other's answers. In the fourth phase (i.e., instruction), the experts are given pencils with attached erasers and are assured that they can revise their answers at any time to further reduce bias (Johnson et al., 2010a). Subsequently, in the fifth phase, the experts answer some background questions about their working experience.

**Figure 3:**
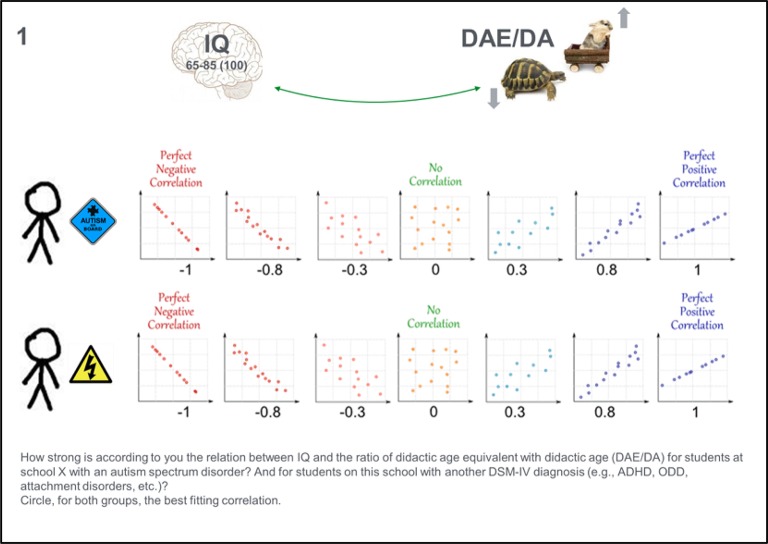
**Material for elicitation question 1: Eliciting a point estimate by selecting the best fitting correlation category for two groups**.

In phase six, the elicitation phase, the facilitator reads the questions aloud and the experts answer the same question simultaneously. It should be stated that experts can discuss their answers together or think out loud. The first task, as a warming up, is for the experts to select the most plausible correlation value from a set of illustrated correlation categories (see Figure 3). The illustrated categories are based on a picture from MathIsFun.com (Pierce, 2014), which is also used in the education phase to explain the concept of correlations. Specifically, in our application the experts received the following question with Figure 3:

“1. How strong do you think the relation between IQ and the ratio of didactic age equivalent with didactic age (DAE/DA) is for students at school X with an autism spectrum disorder?^2^ And for students at this school with another DSM-IV diagnosis (e.g., ADHD, ODD, attachment disorders, etc.)? Circle the best fitting correlation for both groups.”

**Figure 4:**
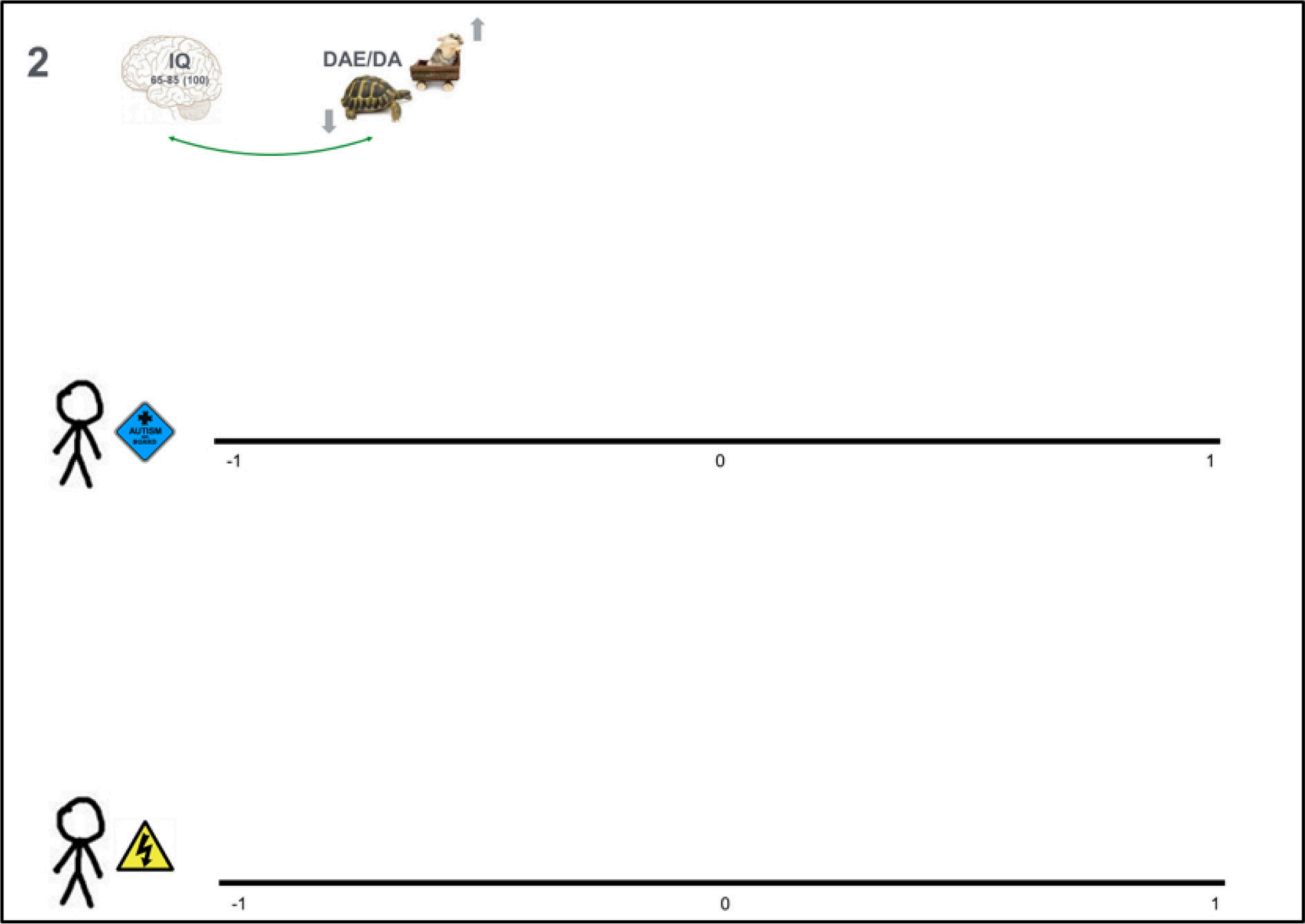
**Material for elicitation question 2: Scale ranging from −1 to +1 on which experts indicate (1) a point estimate, (2) a lower and upper limit, (3) the probability of all values by means of 20 stickers each representing 5%**.

The second question is the trial roulette question. As a first step, experts are asked to indicate the strength of the relationship of interest with a cross on a continuous scale ranging from −1 to +1 (see Figure 4). Specifically, the question in our application was:

“2a. In the previous question you provided an estimate of the relation between IQ and DAE/DA for students enrolled at school X with and without autism spectrum disorder. Indicate with a cross on the A3 paper how strong you think this relation is for both groups when you can choose from all values between −1 and 1.”

Subsequently, they were asked:

“2b. Maybe you are insecure about the estimates you just provided. Indicate on the axis at the previous page also what your lower and upper limit for this estimate would be.”

Finally, the experts receive 20 removable stickers (⊘ = 8 mm), each representing 5% probability, to indicate the plausibility of values between their lower and upper limit. The written instruction they receive is:

“2c. Use the 20 stickers to indicate the weight of your expectation at every place between those limits (further instruction is provided by the facilitator).”

The facilitator explains that stickers can overlap horizontally to represent a very dense distribution. The stickers, however, cannot overlap vertically, because the height of the distribution represents probability, and each sticker represents 5% irrespective of the vertical overlap. The stickered distributions are the target of the trial roulette question, and the main output the elicitation procedure.

The third question is a feedback question to help the experts reflect on their trial roulette responses, and adjust their answers when necessary. The feedback question assesses concordance probability (Gokhale and Press, 1982). When we let *X*_*i*_ denote educational performance of student *i*, and *Y*_*i*_ cognitive potential of student *i*, then concordance probability inquires the probability that *Y*_2_ > *Y*_1_ given that *X*_2_ > *X*_1_. According to Clemen et al. (2000), assessing concordance probability is the second best method to elicit correlations. Specifically, the experts answered the following questions in our application:

“3a. Imagine we select a hundred times two random students with autism spectrum disorder at school X. How often out of hundred do you think that the one with the highest cognitive potential also has the best educational performance?”

“3b. Imagine we select a hundred times two random students with a DSM-IV diagnosis other than autism spectrum disorder at school X. How often out of hundred do you think that the one with the highest cognitive potential also has the best educational performance?”

The experts are asked to disregard previous responses in answering this question to let it function as a proper feedback question. Hence, the relation between concordance probabilities and correlations is not explained to the experts. When every expert has written down their answer, the facilitator asks the experts for their values and translates the values into correlations using:

(1) $$\begin{array}{l} r=\sin\left(0.5\left[\frac{2\pi x}{100}-\pi \right]\right), \end{array}$$

where *r* is the correlation, and *x* is the frequency as provided by the expert. The experts are asked to review and adjust their stickered distributions considering their answers to the concordance probability question. When the experts are satisfied with their judgment distributions, they can continue to the evaluation phase of the elicitation procedure. The questions asked in this phase are specified in the next section.

#### 2.1.3. Elicitation event

The elicitation event took place at a school for youth with behavioral problems where all experts had a meeting scheduled that day. Before the start of the elicitation, all experts gave permission to audio-record the elicitation. The duration of the elicitation event was 40 min.

#### 2.1.4. Assessment of measurement properties

When expert judgments are elicited, validity indicates that the distributions accurately reflect the uncertain knowledge of the experts (Van Lenthe, 1993). In the elicitation procedure, validity is therefore assessed with questions about the elicitation procedure to the experts. More specifically, in our application face validity was assessed with the following question:

“To what degree do you feel that your expert-knowledge about the relation between cognitive potential and educational performance was assessed accurately?”

Not at all / Not really / Neutral / A little bit / Completely

Feasibility is assessed by two statements. The first statement is:

“I thought the questions with their explanations were clear.”

Not at all / Not really / Neutral / A little bit / Completely

The second statement is:

“I thought the questions were easy to answer / conduct.”

Not at all / Not really / Neutral / A little bit / Completely

After each question and statement space is provided to add an explanation. The mean scores over experts were calculated for the two statements, and the average was taken as a final estimate of feasibility. Additionally, the participants answer an open follow-up question:

“Which question did you find the least clear, and why?”

Furthermore, the correlation among individual experts' responses on the trial roulette question and the concordance probability feedback question was computed to assess convergent validity between questions within the procedure. Additionally, the absolute differences between experts' responses on the trial roulette question and the concordance probability feedback question were calculated as another measure of convergent validity. Subsequently, the coherence among experts with respect to the same question was evaluated as an indication of validity, since we expect experts do agree to a certain extent. Finally, a retest was conducted to assess test-retest reliability. All calculations were conducted in R (R Core Team, 2015). Relevant data and R-code are provided as online Supplementary Material (Part III).

### 2.2. Results

The elicitation event proceeded as planned. The experts discussed their views on the population and measures in the clarification phase, and indicated that they understood everything explained in the education phase. During the first question to elicit correlations, the experts discussed the direction of the correlation, and they mentioned that their preferred correlation was not amongst the answer categories. Additionally, they discussed differences among IQ tests. During the second and third question, the experts mainly discussed the task, but not their judgments. One expert varied the vertical distance between stickers substantially, which was noted by the facilitator and adjusted by the expert.

**Figure 5:**
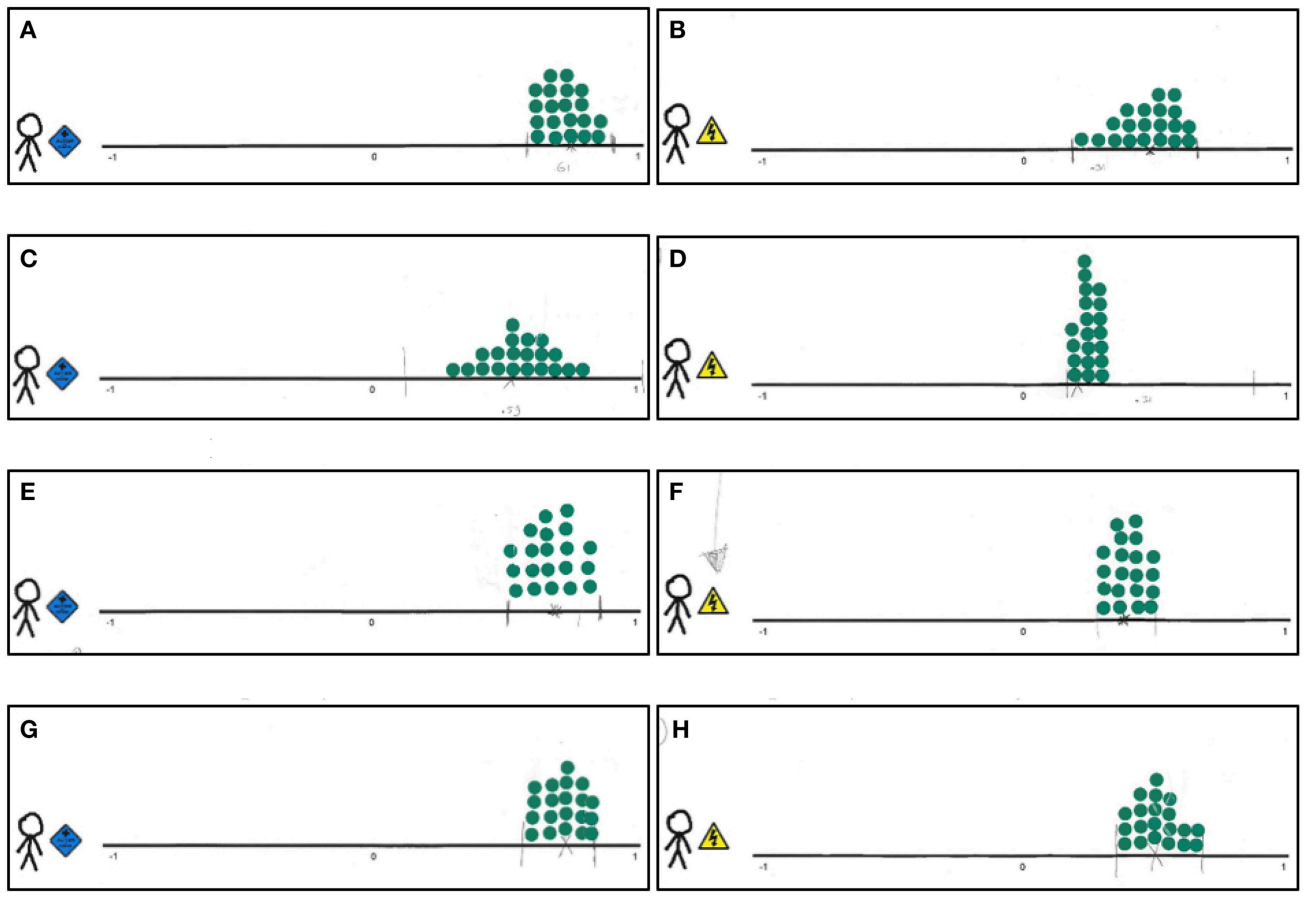
**Trial roulette responses for the correlation between cognitive potential and educational performance for youth with ASD and youth with diagnoses other than ASD enrolled in special education for youth with severe behavioral problems. (A)** Expert 1, youth with ASD. **(B)** Expert 1, youth with diagnoses other than ASD. **(C)** Expert 2, youth with ASD. **(D)** Expert 2, youth with diagnoses other than ASD. **(E)** Expert 3, youth with ASD. **(F)** Expert 3, youth with diagnoses other than ASD. **(G)** Expert 4, youth with ASD. **(H)** Expert 4, youth with diagnoses other than ASD.

Figure 5 shows the elicited distributions for all experts (rows) by evaluated target population (columns), and Table 1 shows the experts' point estimates. The distributions depicted in Figures 5A,C,E,G show that for youth with ASD the correlation between cognitive potential and educational performance is between 0.29 and 0.79 according to expert 2, while the other experts expect the correlation to be 0.5 or higher, up to 0.86. For youth with diagnoses other than ASD (Figures 5B,D,F,H), expert 2 is most specific and expects the correlation to be between 0.16 and 0.31. The other experts are somewhat more uncertain, and expect somewhat higher correlations, but all expect that the correlation for youth with ASD is likely larger than that for youth with other DSM-IV diagnoses.

**Table 1:** **Elicited point estimates and their absolute differences for the correlation derived from question 2a, and question 3 on concordance probability**.

	***r*** **ASD (Q2a)**	***r*** **ASD (Q3a)**	**Δ**	***r*** **no ASD (Q2a)**	***r*** **no ASD (Q3b)**	**Δ**
Expert 1	0.725	0.612	0.112	0.457	0.249	0.226
Expert 2	0.525	0.588	0.063	0.200	0.309	0.109
Expert 3	0.675	0.707	0.032	0.375	0.309	0.066
Expert 4	0.725	0.809	0.084	0.500	0.588	0.088

*Q2a refers to Question 2a where the expert is asked to provide a point estimate for the correlation*.

*Q3a refers to Question 3a which requires the expert to provide a frequency for the concordance probability for youth with ASD*.

*Δ refers to the absolute difference between the two previous columns*.

*Q3b refers to Question 3b which requires the expert to provide a frequency for the concordance probability for youth with diagnoses other than ASD*.

**Figure 6:**
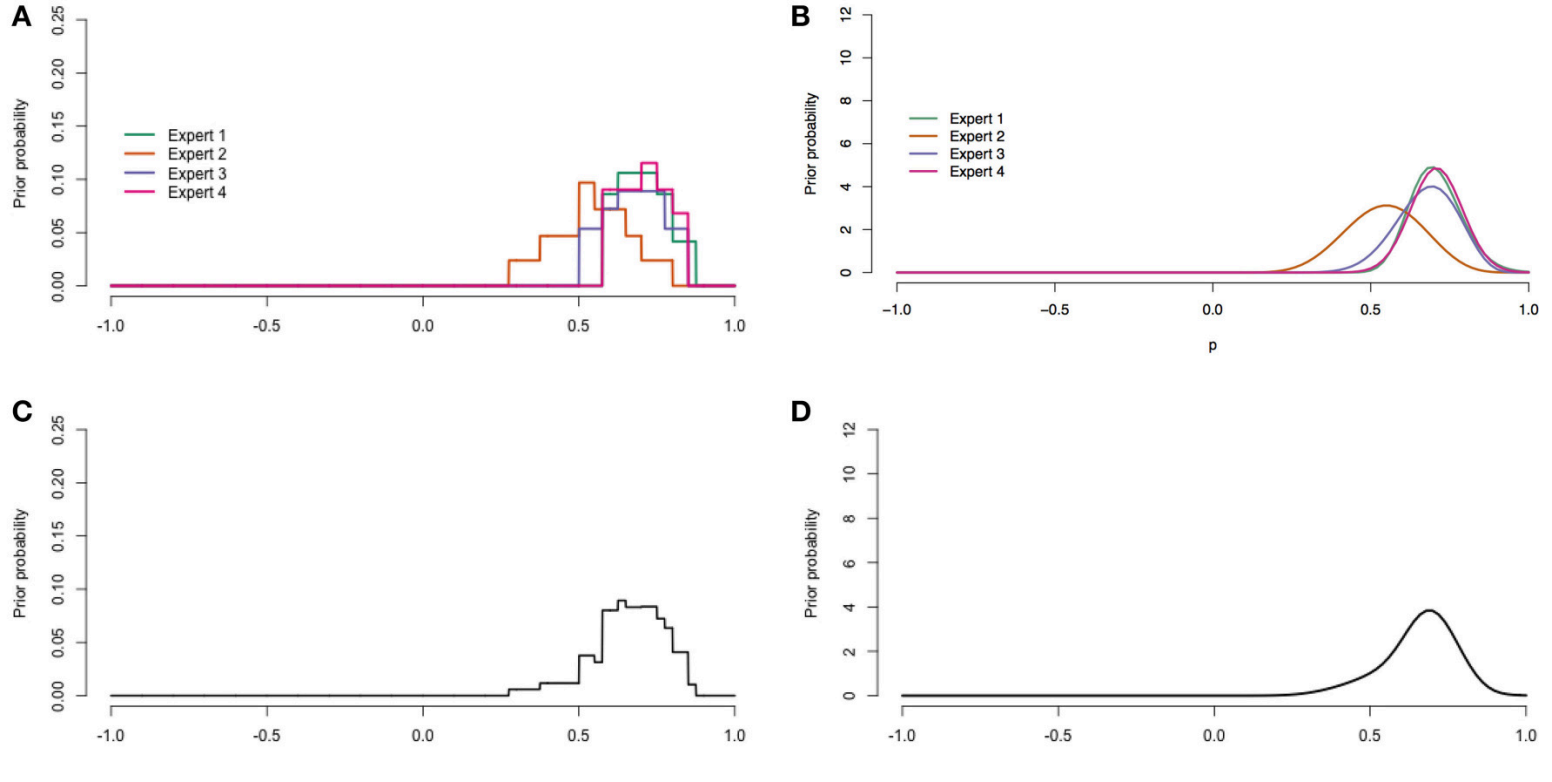
**Digitalized expert judgments for youth with ASD. (A)** Histogram distributions. **(B)** Parametric distributions. **(C)** Pool of histogram distributions. **(D)** Pool of parametric distributions.

**Figure 7:**
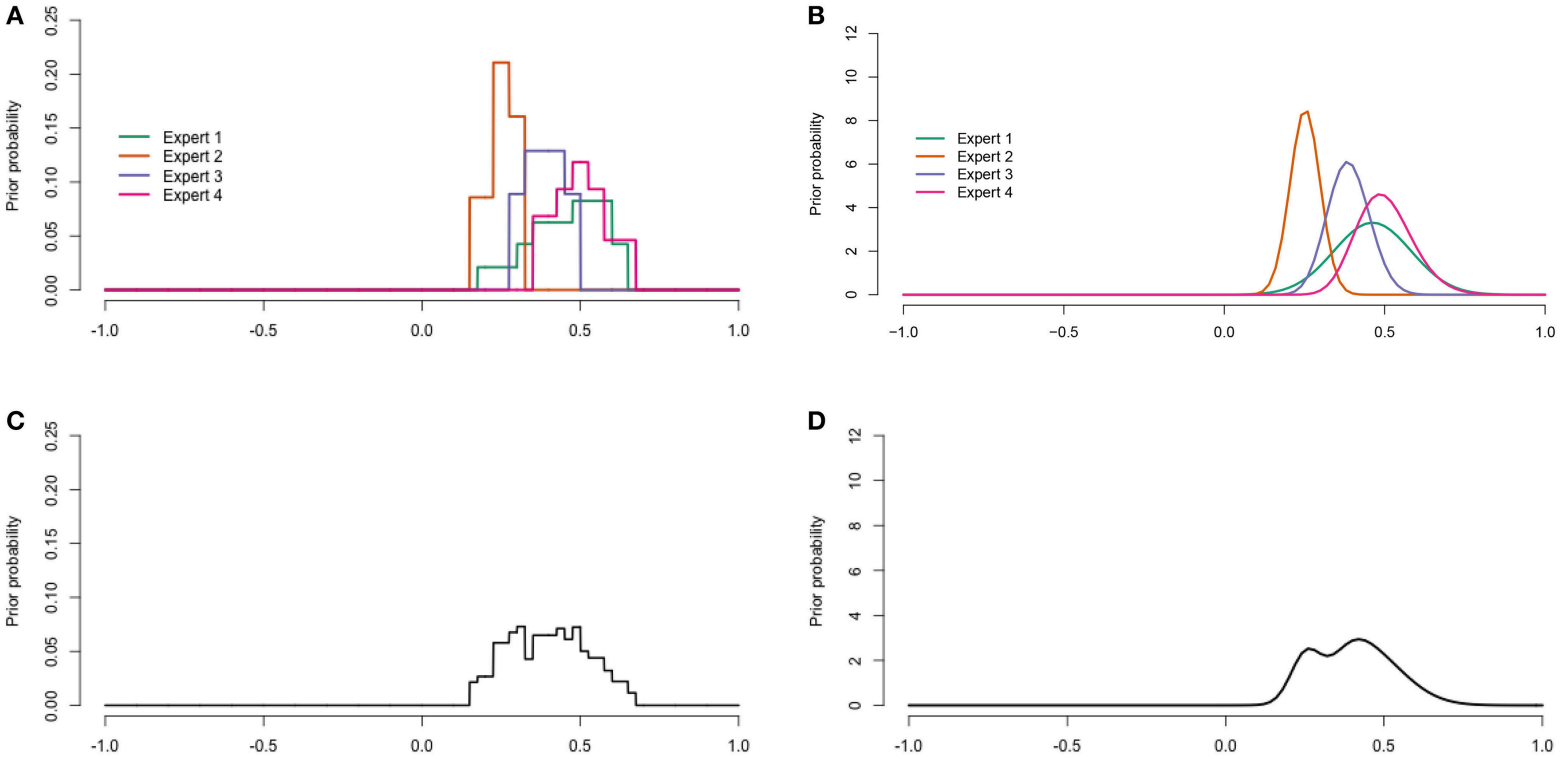
**Digitalized expert judgments for youth with diagnoses other than ASD. (A)** Histogram distributions. **(B)** Parametric distributions. **(C)** Pool of histogram distributions. **(D)** Pool of parametric distributions.

**Figure 8:**
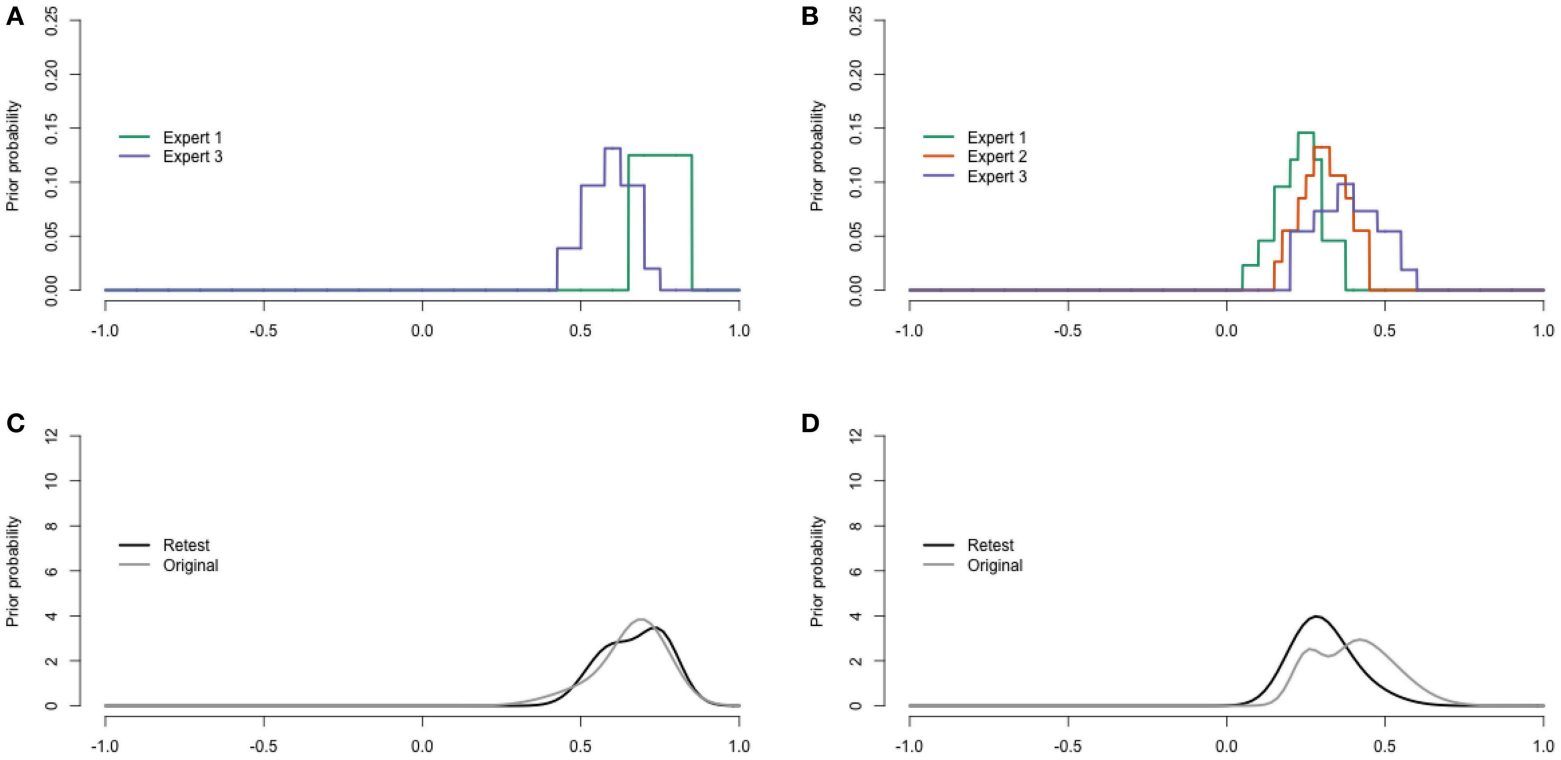
**Digitalized expert judgments retest. (A)** Youth with ASD. **(B)** Youth with diagnoses other than ASD. **(C)** Pool for youth with ASD. **(D)** Pool for youth with diagnoses other than ASD.

The raw data was digitalized after the procedure described in Appendix 2. Figures 6, **8** display the digitalized distributions of the experts in four ways for youth with ASD and youth with diagnoses other than ASD, respectively. Figures 6A, 7A show the distributions as histograms, which can be directly used as priors in a Bayesian analysis (Albert, 2009), but this is not a straightforward option in current software. Another way to process the results is as distributions with a known form; parametric distributions (see Figures 6B, 7B). Parametric distributions can be derived from histogram distributions by means of the Sheffield Elicitation Framework R file (SHELF; Oakley and O'Hagan 2010). Specific code, and the equations for the parametric priors are provided in Appendix 3. Parametric distributions can be used directly as priors in a Bayesian analysis in most Bayesian software. The information provided by the histograms and parametric distributions is similar to that of the raw data as described in the previous paragraph.

The histogram and parametric distributions of the separate experts can also be pooled to obtain an idea of the judgments of the experts as a group. One method to aggregate the distributions is linear pooling (Genest and Zidek, 1986). Linear pooling is a method in which the (weighted) average distribution is calculated. The determination of weights received considerable attention in the literature. For example, experts can be assigned equal weights, experts can be ranked, experts can rank themselves and weights can be attributed proportionally to this ranking, or a performance based method such as the the Classical Model (Cooke, 1991) can be applied (Winkler, 1968). The Classical Model determines weights based on a score for calibration and information. This method requires relevant seed variables for which the truth is or becomes known. In the current study we wanted the prior to reflect the current view of the experts as a group, hence we chose equal weights. The pooled histogram distributions obtained with equal weights are shown in Figures 6C, 7C, and further explained in Appendix 4. Figures 6D, 7D show the pooled parametric distributions. More details on the linear pool of parametric distributions are provided in Appendix 5. For the population with ASD the mode for the correlation is around 0.67, and the 95% interval of values that the experts put most weight on ranges from about 0.41 to 0.86. The population with diagnoses other than ASD does not have one clear mode, but the 95% interval ranges from about 0.18 to 0.64 in both the histogram (Figure 7C) and parametric pooled distribution (Figure 7D).

#### 2.2.1. Validity

The four experts rated face validity with 4, 2, 4, and 4 respectively on a scale from 1 to 5. The expert that provided the lowest score wrote in the open space after the question about the accurateness of the assessment: “More engaged with the statistics → are your own answers reliable? It has to be correct.” The expert's comment was interpreted as indicating that transforming her ideas into proper responses was more difficult than forming judgments, which is not problematic as long as she was satisfied with the final result. The average face validity score of 3.5 was interpreted as satisfactory.

The experts provided scores of 4, 5, and 5 for clarity, and 4, 4, 4, and 5 for ease of of the questions. The average score for feasibility was thus 4.46. The expert that provided the 4 for clarity added that once she had thought about the questions, they were clear. One expert did not provide a score for clarity and added that the verbal explanations were absolutely necessary for her. The feasibility score was interpreted as excellent, because verbal explanations are part of the procedure. Two experts indicated which question they found least clear. One expert wrote that question 1 was the least clear, and explained that this question contained a mistake. Indeed, the question referred to DA/DAE instead of DAE/DA, but this was clarified when the question was addressed, so it will not have affected the validity of the responses. The other expert wrote that question 2 was the least clear, but did not explain her response.

Convergent validity between questions within our procedure was first evaluated by correlating the experts' trial roulette point estimates (Table 1, column 1 and 4), and their answers to the concordance probability question converted to a correlation by means of Equation 1 (Table 1, column 2 and 5). Note that the experts were asked to reconsider their probability distribution after obtaining a correlation value for their concordance probability response, but did not adjust their initial point estimate. With respect to adolescents with ASD, the correlation between the responses to both questions was 0.59, (*SE* = 0.57). The Bayes factor quantifying the relative evidence for a positive correlation vs. a correlation of zero as calculated by JASP 0.8.0.0 (JASP Team, 2016) with default priors was 1.2. With respect to adolescents with other DSM-IV diagnoses, the correlation was 0.42 (*SE* = 0.64), and the Bayes factor was 0.9. The point estimates are an indication of sufficient convergent validity. However, the standard errors show that with four participants the estimates must be interpreted with caution. Additionally, the Bayes factors suggest that there is more evidence for a positive correlation for the first population, but more evidence for a correlation of zero for the second population.

Correlations can be perfect when a bias is systematic, therefore, the absolute difference between the two point estimates may be an even more important indication of convergent validity. The differences between estimates from the trial roulette and concordance probability question are provided in column 3 and 6 of Table 1. Over the two populations, the difference was on average 0.10 (0.07 and 0.12 for the population with and without ASD respectively), which we consider a small difference, and thus a positive indication of convergent validity.

Since the trial roulette method is implemented in the procedure because of the distributions it provides, we also comment on convergent validity between the concordance probability results and the raw distributions (Figure 5). We note that all point estimates given in Table 1 fall within the distributions specified in Figure 5, which means that the point estimates provided in the concordance probability questions are also among the plausible values in the accompanying trial roulette response. These matching responses are a positive indication of convergent validity, but note that participants were allowed to adjust their distributions after receiving feedback from the concordance probability question.

The coherence between the judgment distributions of different experts was taken as a measure of validity. Figure 6A shows that for the population with ASD, the expert judgments clearly cluster and overlap supporting the validity of the procedure. Figure 7A shows that for the population with diagnoses other than ASD the judgments also cluster, but the judgments of expert 2 and expert 4 do not overlap. Since the judgments of expert 2 and expert 4 both overlap with expert 1 and expert 3, it was considered an indication of sufficient validity. To further improve the coherence between expert judgments, the facilitator could encourage the experts to discuss their answers and distributions. The facilitator could, for example ask an expert: “Can you tell me about your distribution and explain the decisions that you have made?”

#### 2.2.2. Reliability

To assess test-retest reliability, the experts were sent the same questionnaire by mail 4.5 months after the original elicitation event. Three out of the four experts were able to respond within 4 weeks. The responses, however, were different than expected: Questions were skipped (expert 2), and the experts (expert 1 and 3) that provided an answer to the concordance probability question for youth with diagnoses other than ASD provided values that correspond to negative correlations, which was inconsistent with their other responses within the retest and original elicitation.

Following Johnson et al. (2010b) the ICC (2,k) of Shrout and Fleiss (1979) was calculated over the point estimates of the three responding experts as a measure of intrarater reliability. The ICC was 0.22 [−0.27, 1.00] with respect to youth with ASD. An ICC value of 0.6 would be moderate. For youth with diagnoses other than ASD, the ICC did not provide sensible values: −1.67 [−4.35, 1.00], because the residual variance was larger than the variance between occasions. Thus, intrarater reliability was insufficient with respect to the point estimates.

Distributions for youth with ASD were only provided by expert 1 and 3 in the retest (see Figure 8A). For youth with other diagnoses, expert 2 stickered a shape instead of a histogram. Nevertheless, we were able to digitalize it in the form of a histogram prior, giving Figure 8B. The pooled retest and original distributions are provided in Figures 8C,D. Despite the inconsistencies in the concordance probability and correlation point estimates, the trial roulette distributions in the retest were similar to the distributions in the original test.

In sum, conventional and custom measures of face-validity, feasibility, convergent validity, and coherence provided positive indications for the validity of the elicitation procedure. The results of the retest were less positive and raise an number of possible interpretations: the poor results for the point estimate reliability and inconsistent concordance probabilities may show that test-retest reliability is low, or that a face-to-face group process is important for consistent responses. The experts may have had difficulties to make time and concentrate on the task in their own environments, making them struggle to conduct tasks that they managed to do in the group setting. The trial-roulette distributions showed better test-retest reliability, although one of the experts did not put the stickers according to the instructions. In the next section, the practical use of the elicitation is illustrated with an empirical application.

## 3. Use of the elicitation procedure

Following Figure 1, the current section provides a full description of the empirical application to illustrate the practical use of the elicitation procedure in a model with the correlation as its key parameter.

### 3.1. Step 1. question

The objective in this application was to update the current knowledge of behavioral scientists working in special education for youth with severe behavioral problems about the correlation between cognitive potential and educational performance for two populations at a specific school in the Netherlands. The populations of interest were (1) youth enrolled in special education, because of severe behavioral problems, who have autism spectrum disorder (ASD), and (2) youth enrolled in this type of special education without ASD but with other DSM-IV diagnoses. Examples of DSM-IV diagnoses that youth in the second population have are oppositional defiant disorder (ODD), attention deficit hyperactivity disorder (ADHD), and attachment problems.

Youth enrolled in special education for reasons of severe behavioral problems are a population that is difficult to recruit, because they are considered vulnerable and are subjected to tests more often than most of them desire. To let these youths participate, informative consent is required from the adolescents themselves as well as a parent or legal guardian in case the adolescent is younger than 16. The files that contained the necessary information for our research, contained personal, and often sensitive information, which increases reluctance to participate. As a result, we expected to gather only a small amount of data. On itself, limited data as obtained in the current application can provide little information, but in combination with expert judgments, it can increase the confidence in current expert views, or indicate that adjustments of these views might be relevant, which can also be an impulse for new research.

Ethical approval for the elicitation procedure, and data collection was given by the Ethics Committee of the Faculty of Social and Behavioral Sciences Utrecht (FETC). Informative consent was obtained from the adolescents. When adolescents were younger than 16 years, informative consent was also obtained from a parent or legal guardian.

Cognitive potential was operationalized as intelligence quotient (IQ) measured with the Wechsler Intelligence Scale for Children (WISC-III; Wechsler 1991). Educational performance was operationalized as the youth's didactic age equivalent divided by didactic age (DAE/DA).

### 3.2. Step 2: elicit expertise

The expert sample is described in Section 2.1.1. The elicitation procedure is described in Section 2.1.2–2.1.4 and Appendix 1.

### 3.3. Step 3: construct priors

In the current section, we explain how we constructed priors for all parameters in the bivariate normal distribution: the correlation, the means of DAE/DA and IQ, and the standard deviations of these variables.

The prior for our key parameter, the correlation, was derived from the experts' trial roulette responses for both populations (see Figure 5 for the raw judgment distributions, and Figures 6, 8 for the digitalized judgment distributions). Since our research goal was to update current expertise, and not expertise specifically related to one expert, we preferred a pooled distribution as a prior. As we show in Appendix Section 6.4, combining a pooled prior distribution with data in one analysis gives a posterior result equal to pooling posteriors of analyses in which each expert's judgment distribution was combined with the data separately. Since the latter approach is more straightforward in software currently available, this approach was adopted in the current study. While the pool of histogram distributions (Figures 6C, 7C) seemed very similar to that of parametric distributions (Figures 6D, 7D), we preferred the pool of parametric distributions because parametric distributions are also more straightforward to deal with in current software, which seems relevant for future users of the procedure.

Priors were also composed for the means (i.e., μ) and standard deviations (i.e., σ) of IQ and DAE/DA. The rational for the prior of μ_IQ_, *p*(μ_IQ_), was based on literature. Expert judgments could have been elicited for the other parameters in the model too, but our experts lacked the time for further elicitation practices. Therefore, we made use of the literature to specify sensible prior distributions for these parameters. Youth who are enrolled in special education because of severe behavioral problems score well below average on IQ. The WISC-III uses the following IQ-score classifications: intellectually deficient, borderline, low average, average, high average, superior, and very superior (Weiss et al., 2006). The borderline class was considered most appropriate for our population. The accompanying IQ scores for this class are 70–79. The rounded class middle of 75.0 was considered a good estimate for the average IQ in our population. A variance of 400.0 (*SD* = 20.0) was chosen to construct a prior distribution with its first quartile at 61.51 and third quartile at 88.49. In addition, the distribution was truncated at the values 45.0 and 145.0, since these values constitute the range of the WISC-III. Thus, the equation for the prior was as follows: *p*(μ_IQ_) ~ *N*(75.0, 400.0)*I*_μ_IQ_∈[45, 145]_.

The rationale for *p*(σ_IQ_) was that the standard deviation of IQ is by definition 15.0 in the population (Prifitera and Saklofske, 1998). A common prior for standard deviations is the gamma prior. The shape and rate parameter of the gamma distribution for the standard deviation of IQ were specified such that the first and third quartile of the distribution were 9.57 and 19.28 respectively (*M* = 15.09). Thus, the equation for the prior was as follows: $p\left(\sigma_{\text{IQ}}\right)\sim \Gamma \left(2.0,\frac{1}{7.5}\right)$.

With respect to *p*_μ_DAE/DA__ we know that youth following special education for reasons of severe behavioral problems generally lag behind, and thus have a DAE/DA below 1.0. As a rough estimate for the average DAE/DA 0.75 was chosen. The variance of the mean was specified to be 0.5. With this specification, the first and third quartile of the prior distribution were 0.27, and 1.23 respectively. The distribution was truncated at 0.0 and 1.5, because more extreme values are naturally impossible to constitute the average for the population of interest. Thus, the equation for the prior was as follows: *p*(μ_DAE/DA_) ~ *N*(0.75, 0.50)*I*_μ_DAE/DA_∈[0.0, 1.5]_.

To our knowledge, no literature exists about σ_DAE/DA_. However, on a scale of 0.0 to 1.5, we considered a standard deviation of 0.36 most likely. A standard deviation of 0.36, namely, would create a 95% confidence interval ranging from 0.04 to 1.46, which constitutes 95% of a normal distribution that ranges from 0.0 to 1.5 with a mean value of 0.75. The shape and rate parameters for the gamma distribution were specified such that the first and third quartile were 0.17, and 0.49 respectively (*M* = 0.36). Thus, the equation for the prior was as follows: *p*(σ_DAE/DA_) ~ Γ(2.0, 5.5).

### 3.4. Step 4: collect new data

We obtained informed consent for 28 adolescents enrolled at a Dutch secondary school for youth with severe behavioral problems to collect information on the research variables of interest from the personal records of the adolescents. For 20 adolescents, the records contained the required data on DSM-IV diagnoses, DAE, DA, and IQ were retrieved from participants' school records. DAE was separately reported for technical reading, reading comprehension, spelling, arithmetic, and vocabulary. An average DAE-score was calculated when scores for at least three of the subjects were available, otherwise, the DAE was regarded missing. When multiple IQ-scores were available, the most recent WISC-III score was included.

**Figure 9:**
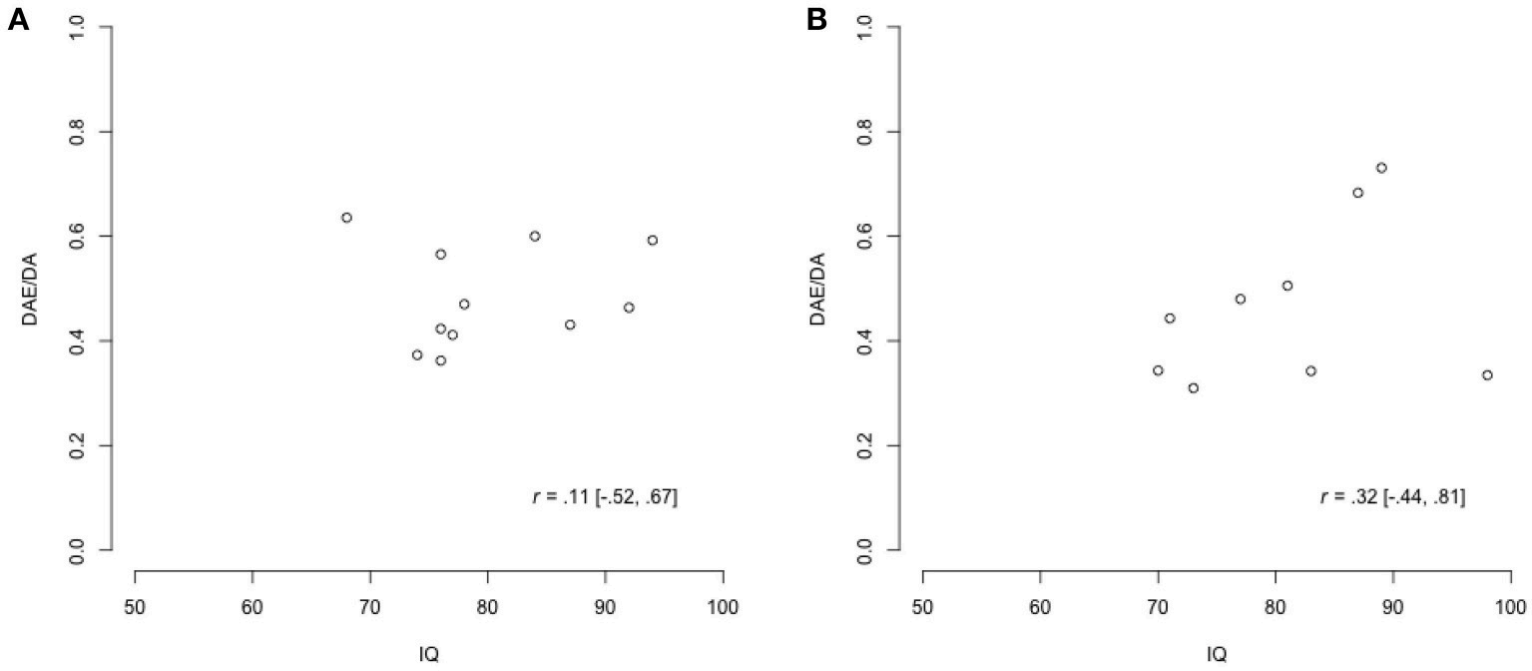
**Scatter plots of the data for DAE/DA and IQ, where *r* indicates the correlation in the data. (A)** Youth with ASD. **(B)** Youth with diagnoses other than ASD.

Eleven out of the 20 adolescents for which sufficient data was present (10 male, 90.9%) belonged to the sample with ASD, and nine (6 male, 66.7%) belonged to the sample with diagnoses other than ASD. The data for DAE/DA and IQ are plotted in Figure 9. As expected, the amount of data was very limited, and would provide little information on the correlations of interest. However, in combination with the expert judgments, it could increase confidence in current expert views or indicate that adjustments of these views are relevant.

### 3.5. Step 5: update

#### 3.5.1. Analysis

In a Bayesian analysis, the prior distribution is multiplied with the (density of the) data, resulting in a posterior distribution. We conducted our analyses with the software JAGS (Plummer, 2013) via the package rjags (Plummer, 2015) in R (R Core Team, 2015). In Appendix 6, we specify the elements of the analyses, and relevant information to properly report a Bayesian analyses (Depaoli and van de Schoot, 2015). Annotated R-code and anonymized data to replicate the results is provided as online Supplementary Material (Part IV).

#### 3.5.2. Results

**Figure 10:**
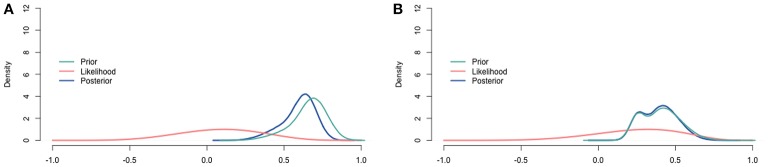
**Visualization of the prior, the relative profile likelihood, and the posterior distribution for the correlation. (A)** Youth with ASD. **(B)** Youth with diagnoses other than ASD.

For the population with ASD, Table 2 summarizes the judgments of the experts, the correlation in the data, and the resulting posteriors. The means of the posterior distributions are all lower than those of the prior distributions as an effect of the low correlation in the data. Another result is that the posterior distributions are more specific than the accompanying priors and the correlation in the data by themselves are. To finish the analysis, we combined the separate posterior distributions for the correlation and constructed the pooled posterior distribution. The pooled posterior distribution for the correlation is displayed in Figure 10A, and summarized in the last column of Table 2. Figure 10A also depicts the aggregated prior, and the (relative profile) likelihood (Bertolino and Racugno, 1992) of the correlation in the data.

**Table 2:** **Elements of the updating procedure: prior per expert, pooled prior, correlation in the data, posterior per expert, and the pooled posterior for the correlation for the population with ASD**.

	***M*** **prior *r* [95% HPD]**	***M*** **pooled prior *r* [95% HPD]**	***M*** **data *r* [95% CI]**	***M*** **posterior *r* [95% HPD]**	***M*** **pooled posterior *r* [95% HPD]**
Expert 1	0.71 [0.55, 0.87]	0.66 [0.40, 0.87]	0.11 [−0.52, 0.67]	0.65 [0.52, 0.78]	0.59 [0.35, 0.79]
Expert 2	0.54 [0.31, 0.78]			0.48 [0.26, 0.68]	
Expert 3	0.68 [0.49, 0.86]			0.60 [0.42, 0.78]	
Expert 4	0.71 [0.55, 0.87]			0.65 [0.51, 0.79]	

*HPD refers to highest probability density. CI refers to confidence interval*.

For the population with diagnoses other than ASD, Table 3 summarizes the judgments of the experts, the correlation in the data, and the resulting posteriors. The means of the posterior distributions are similar to those of the prior distributions, because the correlation in the data is of a similar size as well. Again, the posterior distributions are more specific than the accompanying priors and the correlation in the data by themselves are. As for the population with ASD, we finished the analysis by constructing the pooled posterior distribution. The pooled posterior distribution for the correlation is displayed in Figure 10B, and summarized in the last column of Table 3. Figure 10B also depicts the aggregated prior, and the (relative profile) likelihood (Bertolino and Racugno, 1992) of the correlation in the data.

**Table 3:** **Elements of the updating procedure: prior per expert, pooled prior, correlation in the data, posterior per expert, and the pooled posterior for the correlation for the population with diagnoses other than ASD**.

	***M*** **prior *r* [95% HPD]**	***M*** **pooled prior *r* [95% HPD]**	***M*** **data *r* [95% CI]**	***M*** **posterior *r* [95% HPD]**	***M*** **pooled posterior *r* [95% HPD]**
Expert 1	0.46 [0.22, 0.70]	0.40 [0.18, 0.64]	0.32 [−0.44, 0.81]	0.44 [0.23, 0.66]	0.39 [0.18, 0.61]
Expert 2	0.46 [0.22, 0.70]			0.25 [0.16, 0.34]	
Expert 3	0.39 [0.26, 0.52]			0.39 [0.26, 0.51]	
Expert 4	0.50 [0.34, 0.68]			0.49 [0.34, 0.65]	

*HPD refers to highest probability density. CI refers to confidence interval*.

We investigated the impact of the priors for the standard deviations by means of a sensitivity analysis as advised by Depaoli and van de Schoot (2015) in their checklist for transparent and replicable Bayesian research. The alternative prior that we used was Γ(0.01, 0.01), which is a regular prior for standard deviations. The results show that the posterior distribution is hardly affected by our choice of priors. For the standard deviation of DAE/DA in the population with ASS, the means of the posterior distribution are 0.13 or 0.14 for the regular and informative priors respectively. For the population with diagnoses other than ASS the means of the posterior distributions are 0.18, and 0.19. For the standard deviation of IQ, the means of the posteriors are 10.04 and 10.50 for the regular and informative prior respectively. For the population with diagnoses other than ASS, the means of the posterior distributions are 9.91, and 10.38 for the regular and informative prior respectively.

### 3.6. Step 6: evaluate

The pooled posterior distributions reflect the result of updating the judgments of experts with data. The posterior distributions for both populations are compromises between the prior judgments of the experts and the information in the data. The posterior distributions have smaller 95% intervals than either the pooled prior or the data alone, because our confidence increased by combining the two sources of information. Interesting to note is that the data only slightly affected the posterior distributions for both populations. This small impact is caused by the limited amount of information that can be derived from 11 or 9 data points. The relatively flat and wide likelihood distributions (Figure 10) illustrate this nicely.

According to the updated state of knowledge, the correlation between cognitive potential and educational performance is most likely large for youth with ASD who are enrolled in special education because of severe behavioral problems. By updating the expert judgments with new data, the judgment about the correlation has been slightly modified downwards. This modification raises the question whether additional data would again have such an effect. A new research cycle may be started based on this question. With respect to youth with diagnoses other than ASD, updating the expert judgments with new data slightly modified, but mainly reinforced current expert views of a medium correlation between cognitive potential and educational performance. New data and new experts may further update this adjusted judgment.

Following the research cycle, the school in question gained insight into the views of school psychologists with respect to the relation between educational performance and cognitive potential for two of the populations that visit the school, and the fusion of these views with local data. A new research cycle may be started to further update the current state of knowledge.

## 4. Discussion

The purpose of the current paper was to evaluate and apply a procedure to elicit judgments for correlation priors. The results of this procedure using the trial roulette method are promising. Measures of face-validity, feasibility, convergent validity, coherence, and intrarater reliability showed positive results. Furthermore, the results of the procedure were useful as prior information in a Bayesian analysis.

The proposed elicitation procedure can be used to elicit experts' prior judgments about Pearson's product moment correlations for bivariate models. For models with more variables, conditional correlations need to be elicited to retain a positive definite correlation matrix. Further research is required to see if the trial roulette method is also suited to elicit the conditional correlations. The elicitation of conditional correlations increases in complexity as the size of the correlation matrix increases. Werner et al. (2016) wrote a review on expert judgment for dependence that offers guidance on making choices about summaries of expert knowledge for multivariate distributions.

Several digital trial roulette elicitation tools have been developed. For example, SPIES (Haran and Moore, 2014), and the MATCH Uncertainty Elicitation Tool (Morris et al., 2014). Advantages of these elicitation tools are that they can be easily distributed, and there is no need to digitalize the elicited responses anymore. On the other hand, the digital mode is less suitable for discussion among experts, and providing additional explanation when necessary. Since correlations are considered more complex than probabilities, an interactive (face-to-face) education phase may be more important in this context. Given that experts in our study skipped questions and ignored instructions outside the group setting, we expect that the suitability of digital elicitation differs between populations of experts.

People tend to vary their responses depending on the specific “anchors” (i.e., fixed values) they are provided with (O'Hagan et al., 2006). To avoid too much influence on the judgment process from “random” values, we chose to provide only three tick labels at meaningful points (−1, 0, 1) along the scale of the trial roulette question. A potential issue raised by one of the reviewers, however, was that not providing more tick labels may have lowered the validity of the trial roulette question, since experts may have been unable to pinpoint specific values along the line. Further research is required to investigate whether it is important for valid responses that experts know to what correlation value the points along the axis correspond. If it is important for experts to have more tick labels, it should be investigated how many tick labels are required, and whether they should be evenly distributed along the scale, or be placed at meaningful values like Cohen's (1988) indications of small, medium, and large correlations. A potential increase in validity by placing tick labels should be balanced with the loss in validity that could be induced by anchoring.

The evaluation of the elicitation procedure and the illustrative application have limitations. Most importantly, only four experts participated in the final elicitation procedure. Four experts can be sufficient, but a panel of about eight is recommended (Cooke and Goossens, 1999). When more experts are involved, it is easier to recognize the general opinion and the final result is less sensitive to the misjudgment of one expert. Furthermore, the identification and selection of experts generally is a process with multiple stages in which potential experts are asked to identify other experts until no new names appear. Subsequently, experts are selected based on relevant criteria. In some cases a panel may be installed to select experts based on their curriculum vitae (Cooke and Goossens, 1999). In the illustrative application of the elicitation procedure, one key informant identified and selected experts, which may limit the diversity of the expert's judgments.

Because validity is the accurate representation of experts' judgments in our research context, and our research data was not necessarily unbiased, we did not validate the accuracy of the expert judgments against data. Consequently, we cannot rule out that all experts were wrong about the truth in the population. When finding the truth about the correlation in the population is the main goal, researchers need sufficient unbiased data, or a seed variable that can indicate the accuracy of the experts' judgments (Cooke, 1991).

Considering the distributions of the experts, one might suspect overoptimism (i.e., expecting the effect to be larger than it is in reality) and overconfidence (i.e., specifying too narrow intervals) to play a role. Goldstein and Rothschild (2014), however, showed that even laymen can properly retrieve underlying population distributions about frequencies. Overoptimism can also be reduced by pooling over experts (Johnson et al., 2010a) as we did in the current study. Additionally, the feedback by the concordance probability question can help experts to detect potential overoptimism. Overconfidence may very well be an issue in the experts' judgments. SPIES has shown to reduce overconfidence compared to directly asking for intervals or fractiles, but even in this method 90% intervals cover the truth in only 73.8% of the cases (Haran and Moore, 2010). It may be worthwhile to introduce extra variance in prior distributions based on expert elicitation before updating it with data when trying to retrieve the correlation in the population.

For future use of the elicitation procedure, naturally, the variables and accompanying illustrations should be adjusted to the research questions at hand. Additionally, we would advise to ask experts to reflect on their judgments. Such a reflection creates an additional feedback moment and encourages experts to discuss their judgments, which further promotes judgment synthesis. Directions to facilitate a group discussion on expert judgments have been provided recently in SHELF 3.0 (Oakley and O'Hagan, 2016). Finally, we did not deviate from the way Johnson et al. (2010b) asks the experts about upper and lower limits. Consequently, like Johnson et al. (2010b) we are not certain whether the experts interpreted the limits of their plausible estimate as a 90, 95, 100%, or another confidence interval. Oakley and O'Hagan (2016) provide an instructional slideshow to explain the meaning of plausible limits to experts that can be used in future applications.

With the elicitation procedure, users can progress from having no expert judgments about the correlation at all, to distributions of probable values according to experts, which can be further updated with new data. When the expert judgments and data are alike, the updated distribution shows that experts can increase their confidence. When the expert judgments and data are more dissimilar, the expert views can be adjusted when both sources of information seem trustworthy, but it can also be an important impulse for further research. Thus, combining expert judgments with data either leads to more confident conclusions, or results in new research questions which can be further investigated according to the research cycle.

## Author contributions

MZ and RV mainly contributed to the study concept and design. Development of the expert elicitation questionnaire was performed by MZ, RV, WV, and KL. The pilot test of the expert elicitation questionnaire was directed by MZ with support from KL, and facilitated by RV. Expert elicitation was performed by MZ, and facilitated by WV. Data was analyzed and stored by MZ. HH proposed, among other things, to pool posterior distributions as an alternative of updating the pooled prior, and closely monitored Appendix 2 and 6. MZ wrote and revised the paper with feedback of RV and HH.

## Funding

The first (MZ) and fourth author (HH) are part of the Consortium on Individual Development (CID). The CID is funded through the Gravitation program of the Dutch ministry of Education, Culture, and Science and the Netherlands Organization for Scientific Research (NWO Gravitation 024-001-003). The third author (KL) received a talent grant from NWO: NWO Talent 406-15-062. In addition, the last author (RV) obtained a Vidi grant from NWO: NWO Vidi 452-14-006.

### Conflict of interest statement

The authors declare that the research was conducted in the absence of any commercial or financial relationships that could be construed as a potential conflict of interest. The reviewer AH and the handling Editor declared their shared affiliation, and the handling Editor states that the process nevertheless met the standards of a fair and objective review.

## Footnotes

^1^For an introduction to Bayesian statistics for social scientists we recommend Gill (2014), Kaplan (2014), and Van de Schoot et al. (2013).

^2^The experts received the name of the school of interest, but for privacy reasons the name of the school is not published.
