# Supplementary material for: Application and Evaluation of an Expert Judgment Elicitation Procedure for Correlations
Source: Front Psychol. 2017 Jan 31;8:90. doi: 10.3389/fpsyg.2017.00090 (PMC5282462; doi:10.3389/fpsyg.2017.00090)
Supplement: Supplementary file 1 [file Part_II_original_material.PDF]

Introductie

- Horizon onderzoek: “Project Bayesiaanse updating IQ”

- Subproject met doel:
  (1) Onderzoeken hoe expert-kennis meegenomen kan worden in een data-analyse
- Expert = iemand die kennis heeft of kan bedenken over het onderwerp
- Kennis = kennis, maar er is ook ruimte voor onzekerheid!
  De expert kennis die we willen verzamelen moet vertegenwoordigen hoe zeker/onzeker jullie zijn.
  (2) Evalueren van onze vragenlijst

- **Vragen mogen altijd gesteld worden**
- **Antwoorden mogen altijd aangepast worden**

Onderzoeks doelgroep

Jongeren zoals op het Schreuder College – locatie Villeneuvestraat:

- 12-18 jaar
- Ernstige gedragsproblemen, stoornis in autsime spectrum, kwetsbare leerlingen die in groep gemakkelijk agressief worden
- Laag IQ (gem 65-85, enkele uitschieter naar 100)

DLE / DL

DL =	Didactische leeftijd in maanden (10 per schooljaar vanaf groep 3)
DLE =	Didactisch leeftijds equivalent<br>gebaseerd op technisch lezen, begrijpend lezen, spelling, rekenen en woordenschat
**DLE/DL** =	Verhouding van het geleerde (in maanden) ten opzichte van het ontvangen onderwijs (in maanden) waarbij lager dan 1 wijst op een achterstand, 1 wijst op precies op schema en hoger dan 1 wijst op een voorsprong.

Voorbeeld

DL =	55 (halverwege groep 8)
DLE =	48 (eind groep 7)
DLE/DL =	48/55 = 0.87 (leerachterstand van 13%)

Correlatie

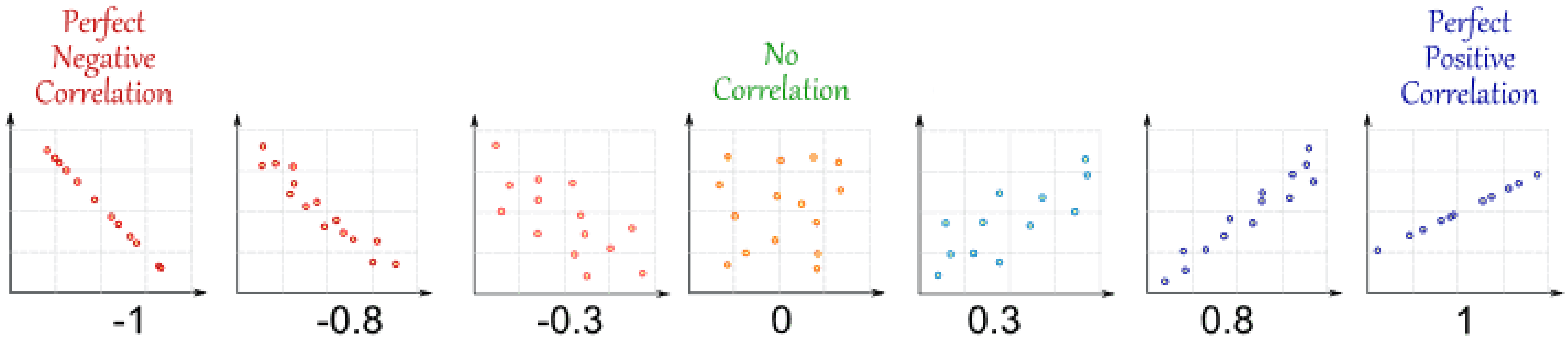

De waarde bepaalt hoe goed de samenhang is (niet hoe steil de lijn is!).

Universiteit Utrecht

Introductie
Deze vragenlijst betreft uw kennis en ideeën over het verband tussen IQ en DLE/DL voor jongeren op het speciaal onderwijs (cluster 4) met en zonder een autisme spectrum stoornis.
Deze kennis zullen we met verschillende methodes meenemen in een data-analyse, waardoor we een beter beeld hopen te krijgen van wat er mogelijk is met de informatie van experts over een onderzoeksvraag.
De vragenlijst begint met een aantal algemene vragen. Vervolgens worden er zes inhoudelijke vragen gesteld. Tot slot volgt er een korte evaluatie.

Algemene vragen

Geslacht …

Leeftijd … jaar

Aantal jaar ervaring als gedragswetenschapper … jaar

Werkzaam op een SO / VSO / EC (doorstrepen wat niet van toepassing is)

Werkzaam met leerlingen op basisonderwijs / praktijkonderwijs / vmbo / havo / vwo niveau (doorstrepen wat niet van toepassing is, meerdere antwoorden mogelijk)

Opmerkingen/toevoegingen

____

____

Participantnummer: ___

1

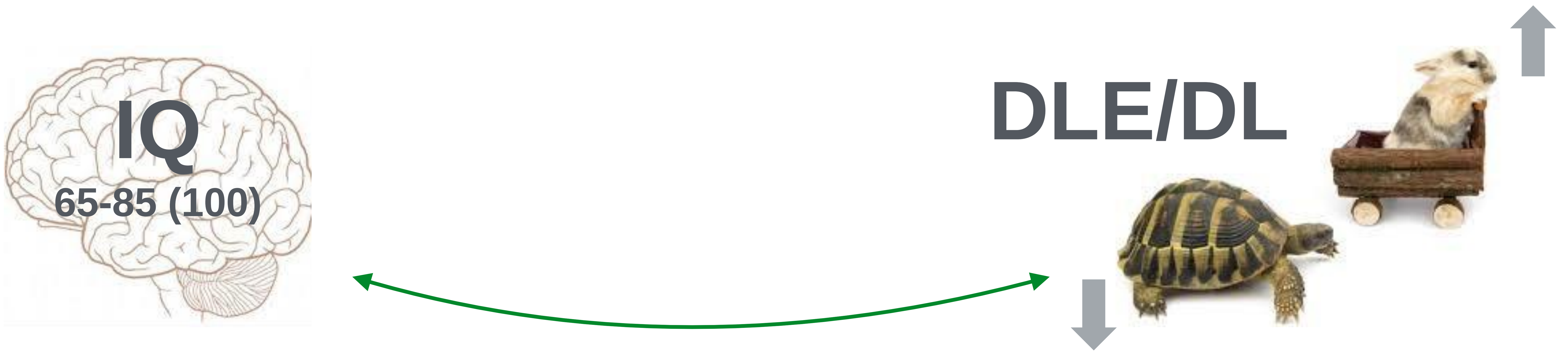

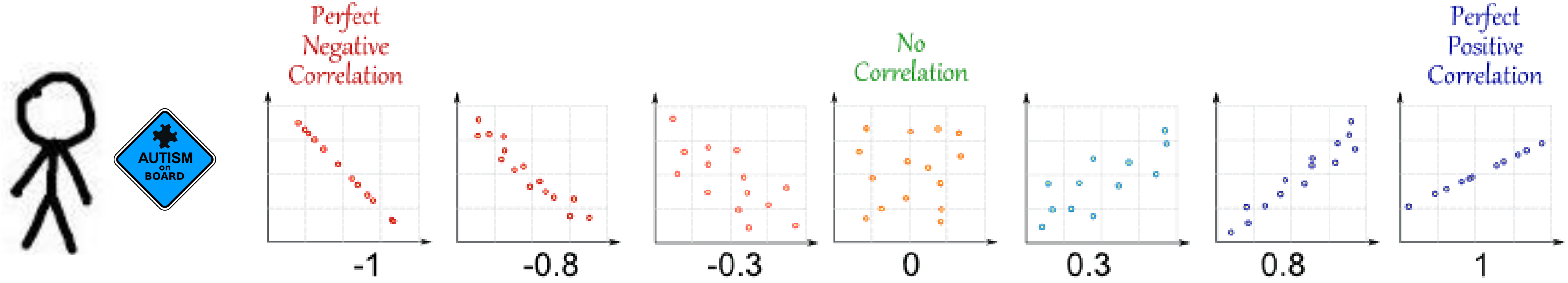

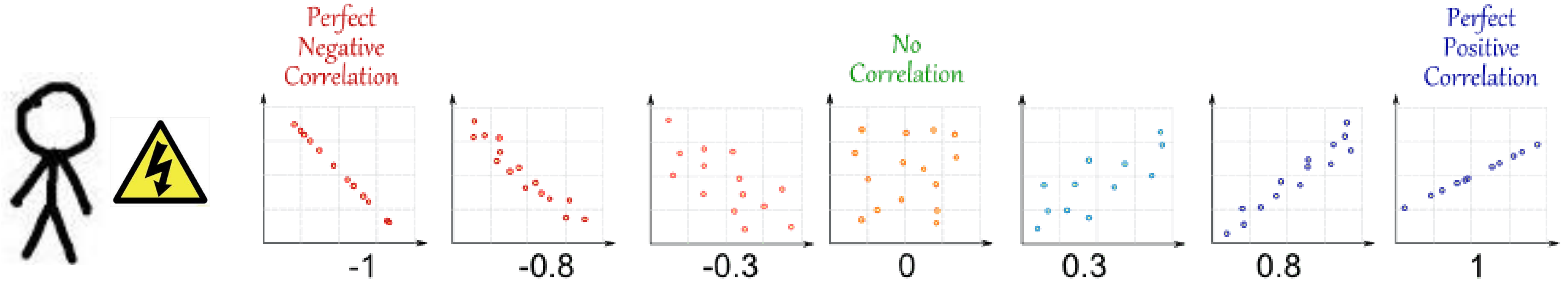

Hoe sterk is volgens jou het verband tussen IQ en de ratio van didactisch leeftijdsequivalent en didactische leeftijd (DLE/DL) voor leerlingen op het Schreuder College met een autisme spectrum stoornis? En voor kinderen op deze school met een andere DSM-IV diagnose (bijv. ADHD, ODD, hechtingsstoornis etc.)?

Omcirkel voor beide groepen de best passende correlatie.

2

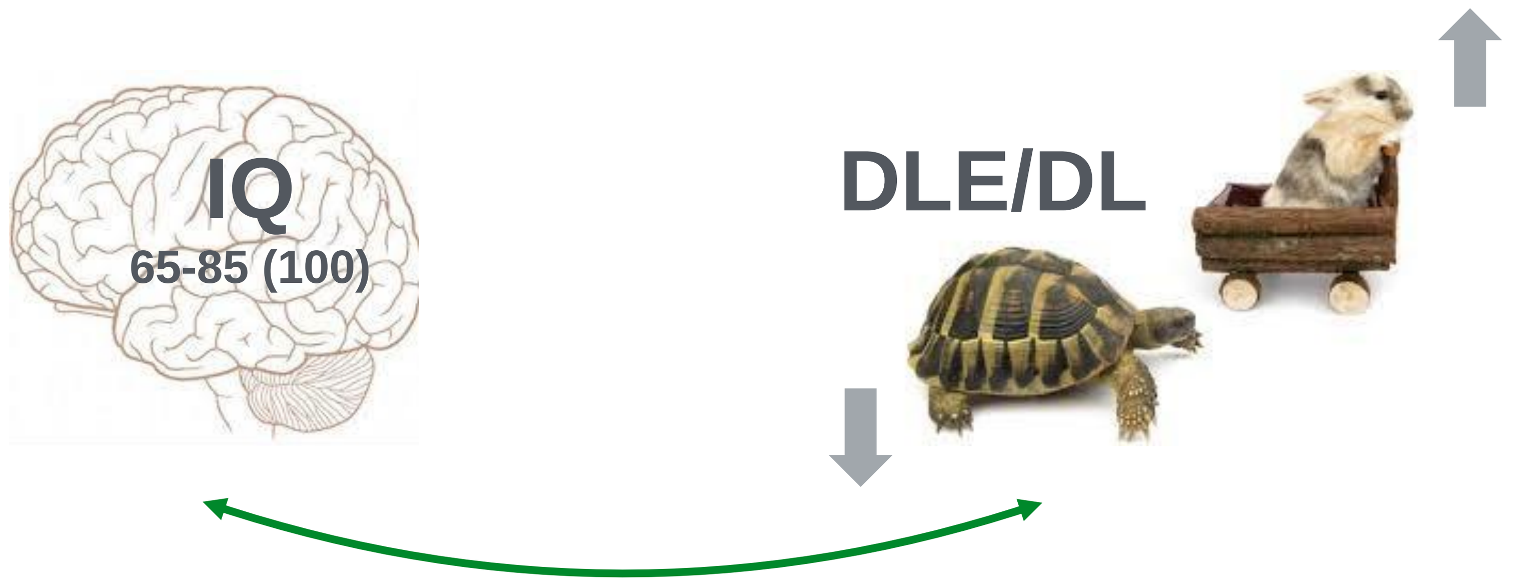

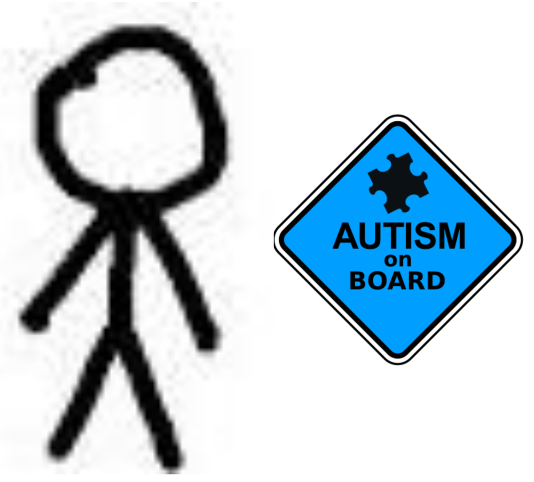

-1 0 1

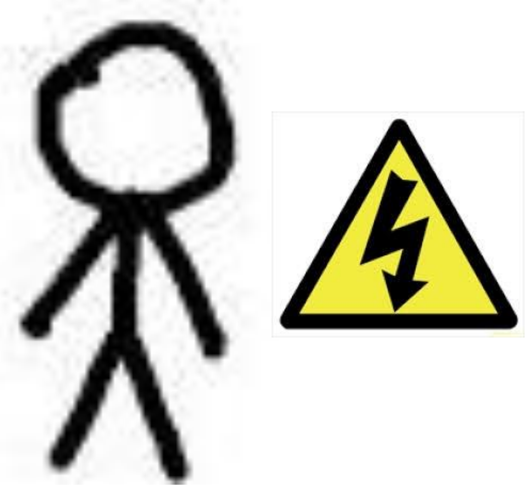

-1 0 1

2a

In de vorige vraag heeft u al een schatting gemaakt voor het verband tussen IQ en DLE/DL voor jongeren van het Schreuder College met en zonder autisme spectrum stoornis.
Geef nu met een kruisje op de schalen per groep op het A3 papier ook aan hoe sterk u denkt dat dit verband is voor de beide groepen als u uit alle waarden tussen -1 en 1 kunt kiezen.

2b

Misschien bent u onzeker over de zojuist gegeven schattingen.
Geef op de lijn op de vorige pagina daarom ook aan wat uw ondergrens en bovengrens voor de schatting zou zijn.

2c

Geef vervolgens met de 20 stickers aan wat het gewicht van uw verwachting is op elke plek binnen deze punten (verdere Instructie wordt gegeven door test-afnemer).

3a

Stel: we selecteren 100 keer twee willekeurige personen met autisme spectrum stoornis het Schreuder college. Hoeveel van de 100 keer denkt u dat de persoon met het hoogste IQ ook het hoogste DLE/DL heeft?

3b

Stel: we selecteren 100 keer twee willekeurige personen met een andere DSM-IV diagnose dan autisme spectrum stoornis van het Schreuder college. Hoeveel van de 100 keer denkt u dat de persoon met het hoogste IQ ook het hoogste DLE/DL heeft?

Universiteit Utrecht

Evaluatie
We willen onze methode om expert kennis te verzamelen graag evalueren, daarom vragen we u om de volgende vragen te beantwoorden.

In welke mate heeft u het gevoel dat de vragen uw expert-kennis op het gebied van het verband tussen IQ en DLE/DL goed hebben gemeten?
Helemaal niet / niet echt / neutraal / een beetje / helemaal

Toelichting: ………

Ik vond de vragen met toelichting duidelijk.
Helemaal niet / niet echt / neutraal / een beetje / helemaal

Toelichting: ………

Welke vraag vond u het minst duidelijk en waarom?

……......................

Ik vond de vragenlijst makkelijk in te vullen / uit te voeren.
Helemaal niet / niet echt / neutraal / een beetje / helemaal

Toelichting: ………

Opmerkingen/toevoegingen

……......................

……......................

Hartelijk bedankt voor uw medewerking!
