# Supplementary material for: Application and Evaluation of an Expert Judgment Elicitation Procedure for Correlations
Source: Front Psychol. 2017 Jan 31;8:90. doi: 10.3389/fpsyg.2017.00090 (PMC5282462; doi:10.3389/fpsyg.2017.00090)
Supplement: Supplementary file 2 [file Part_II_presentation.PPTX]

## Slide 1
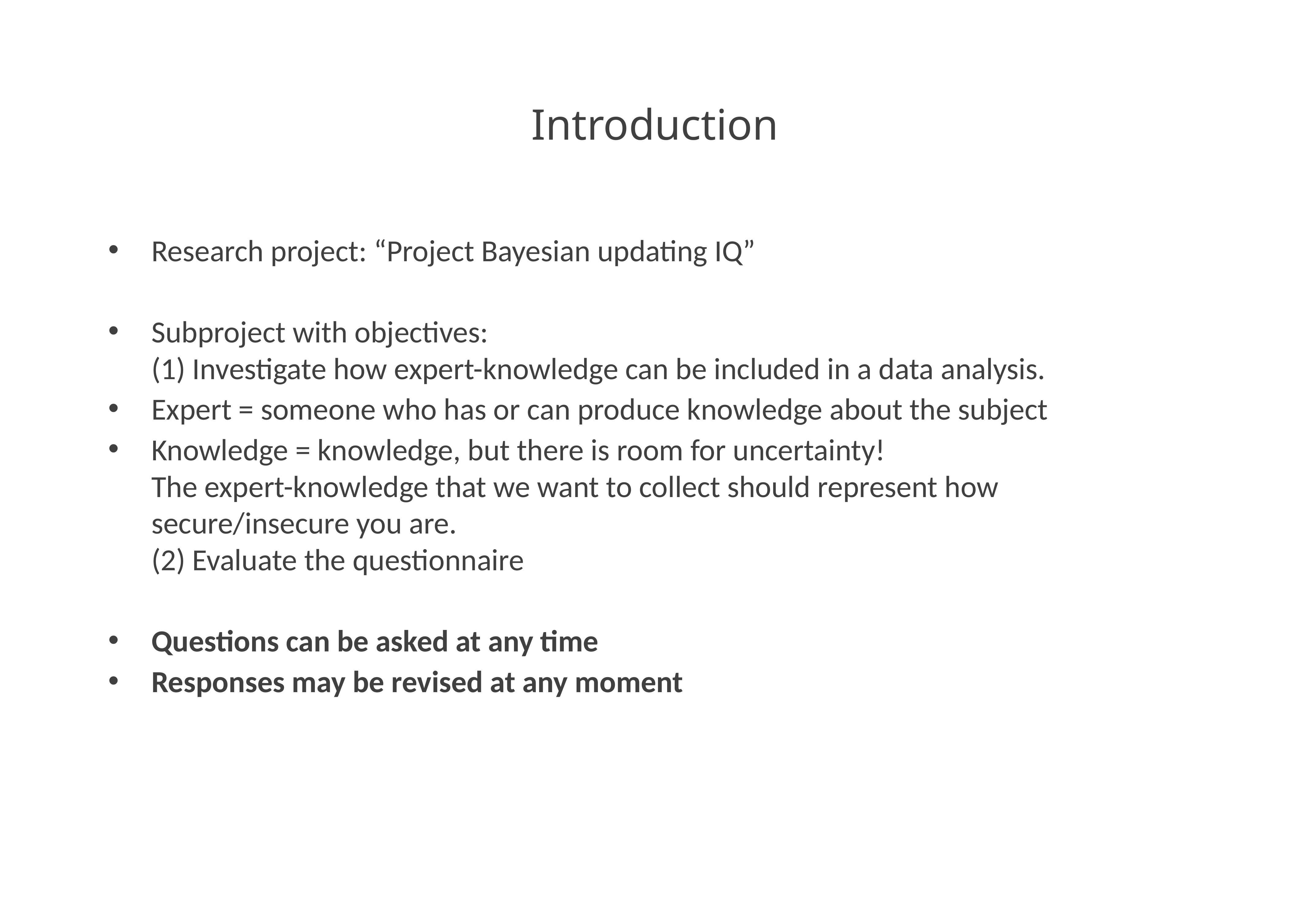

Introduction
Research project: “Project Bayesian updating IQ”
Subproject with objectives:
(1) Investigate how expert-knowledge can be included in a data analysis.
Expert = someone who has or can produce knowledge about the subject
Knowledge = knowledge, but there is room for uncertainty!
The expert-knowledge that we want to collect should represent how
secure/insecure you are.
(2) Evaluate the questionnaire
Questions can be asked at any time
Responses may be revised at any moment

## Slide 2
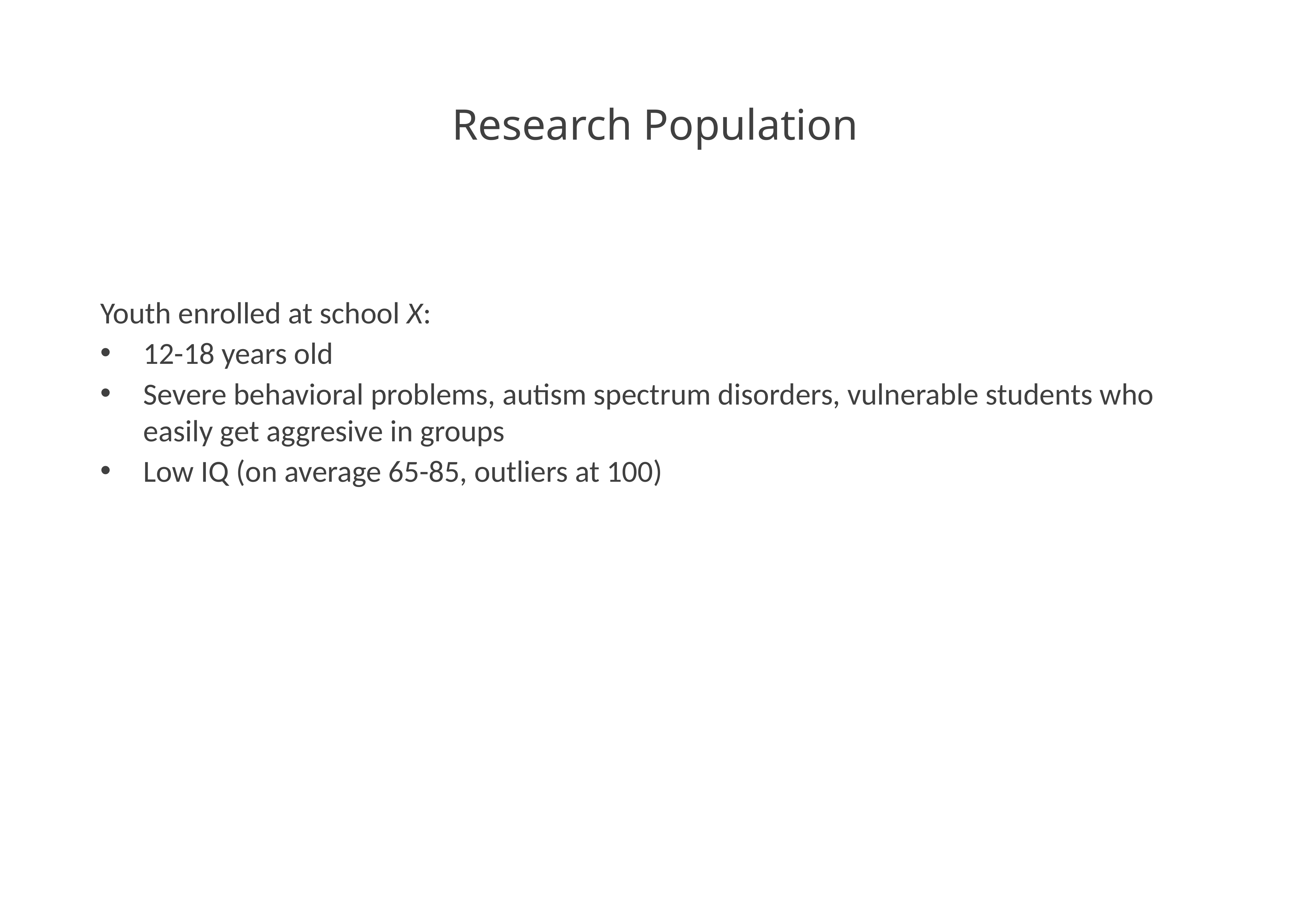

Research Population
Youth enrolled at school X:
12-18 years old
Severe behavioral problems, autism spectrum disorders, vulnerable students who
easily get aggresive in groups
Low IQ (on average 65-85, outliers at 100)

## Slide 3
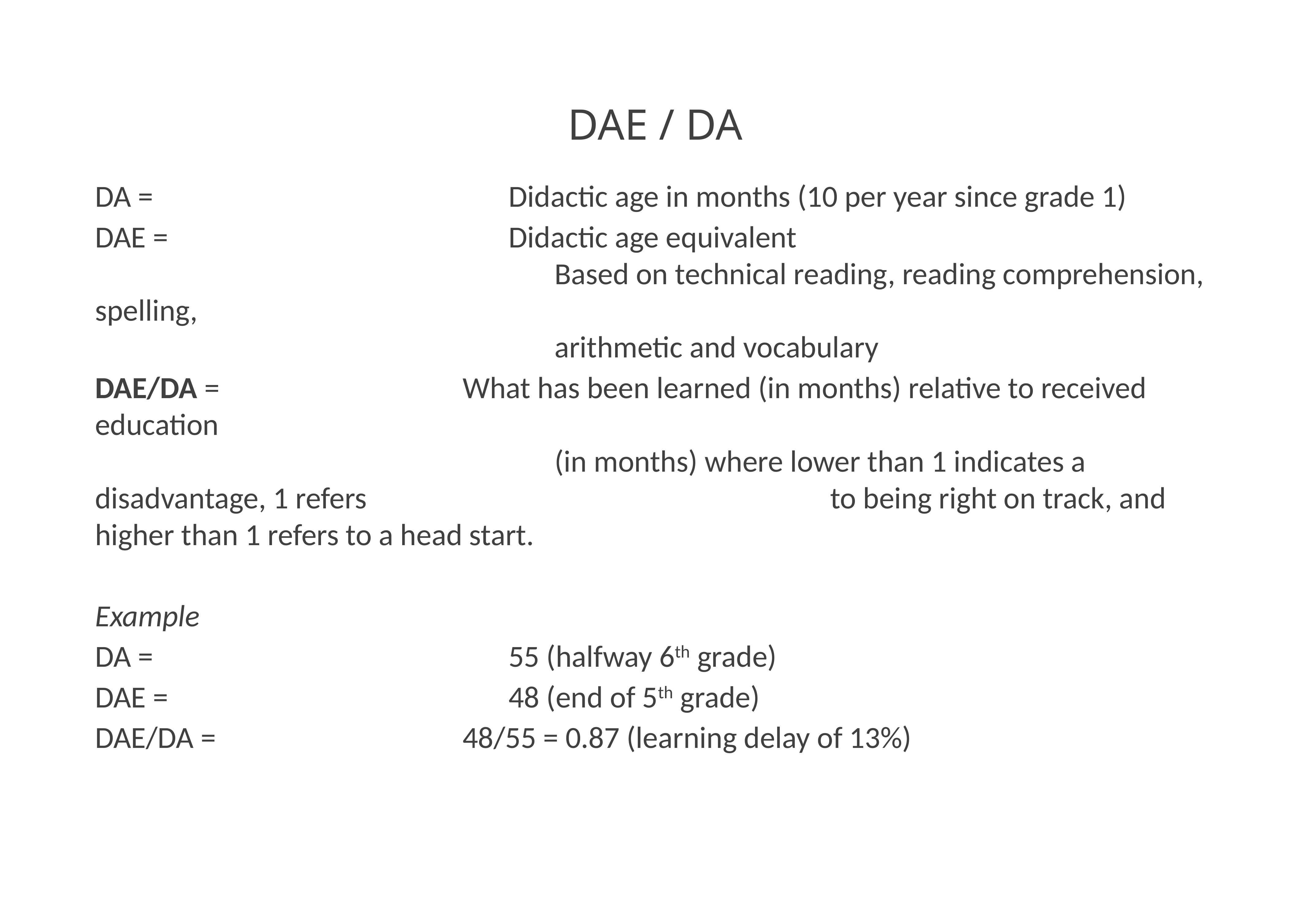

DAE / DA
DA = 								Didactic age in months (10 per year since grade 1)
DAE = 								Didactic age equivalent
										Based on technical reading, reading comprehension, spelling,
										arithmetic and vocabulary
DAE/DA =						What has been learned (in months) relative to received education
										(in months) where lower than 1 indicates a disadvantage, 1 refers											to being right on track, and higher than 1 refers to a head start.
Example
DA = 								55 (halfway 6th grade)
DAE = 								48 (end of 5th grade)
DAE/DA = 						48/55 = 0.87 (learning delay of 13%)

## Slide 4
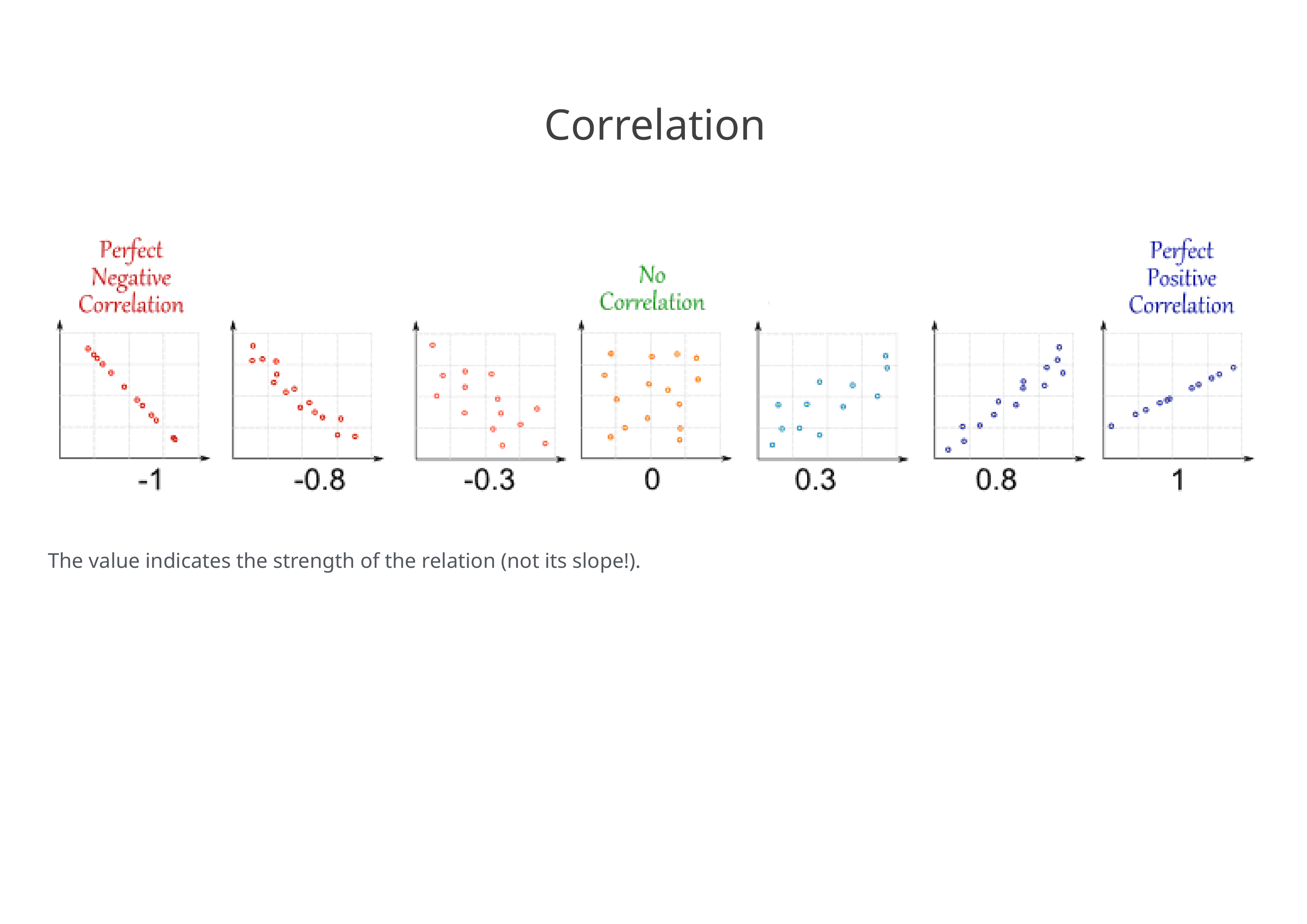

Correlation
The value indicates the strength of the relation (not its slope!).

## Slide 5
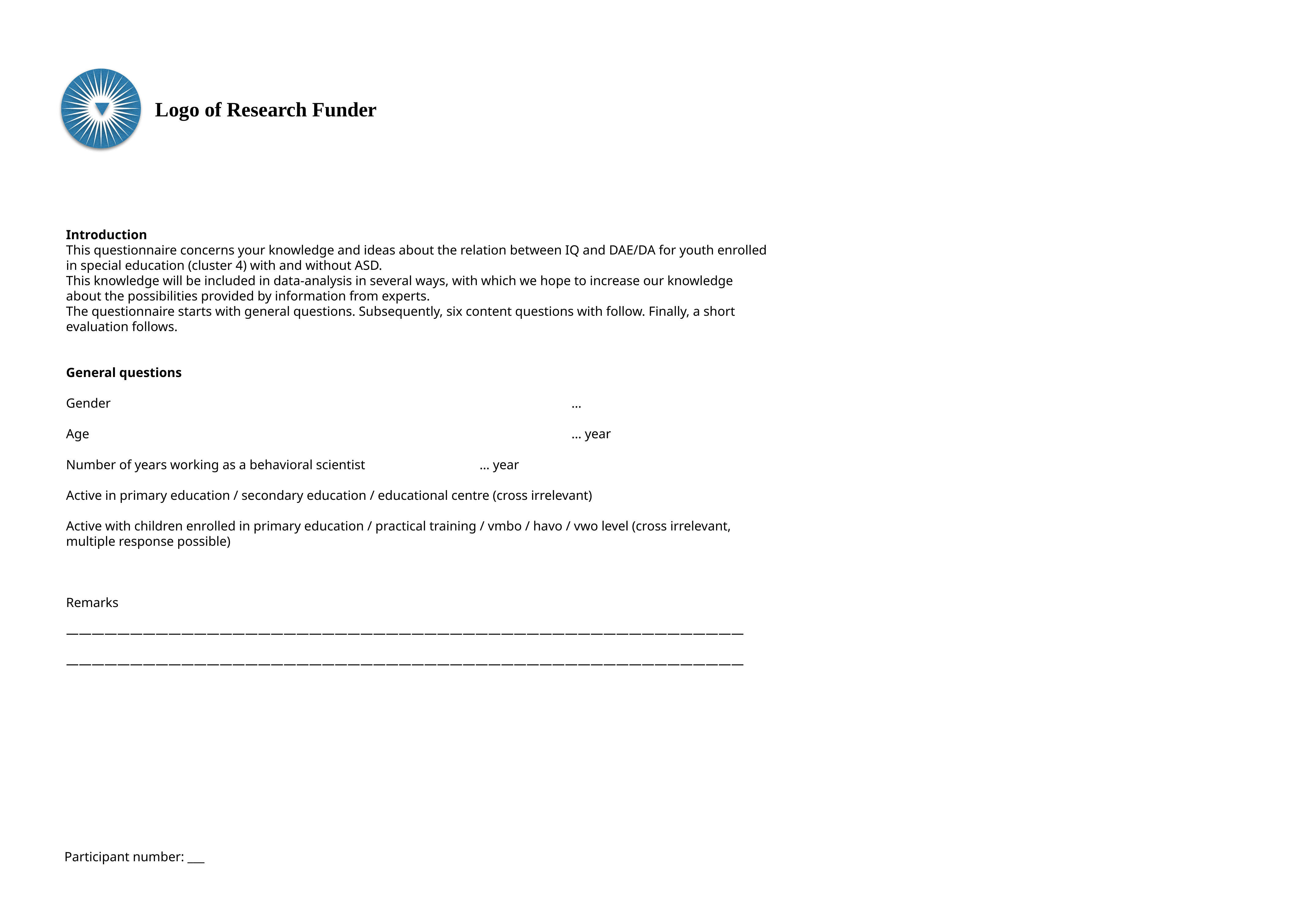

Logo of Research Funder
Introduction
This questionnaire concerns your knowledge and ideas about the relation between IQ and DAE/DA for youth enrolled in special education (cluster 4) with and without ASD.
This knowledge will be included in data-analysis in several ways, with which we hope to increase our knowledge about the possibilities provided by information from experts.
The questionnaire starts with general questions. Subsequently, six content questions with follow. Finally, a short evaluation follows.
General questions
Gender											…
Age 											… year
Number of years working as a behavioral scientist			… year
Active in primary education / secondary education / educational centre (cross irrelevant)
Active with children enrolled in primary education / practical training / vmbo / havo / vwo level (cross irrelevant, multiple response possible)
Remarks
—————————————————————————————————————————————————————
—————————————————————————————————————————————————————
Participant number: ___

## Slide 6
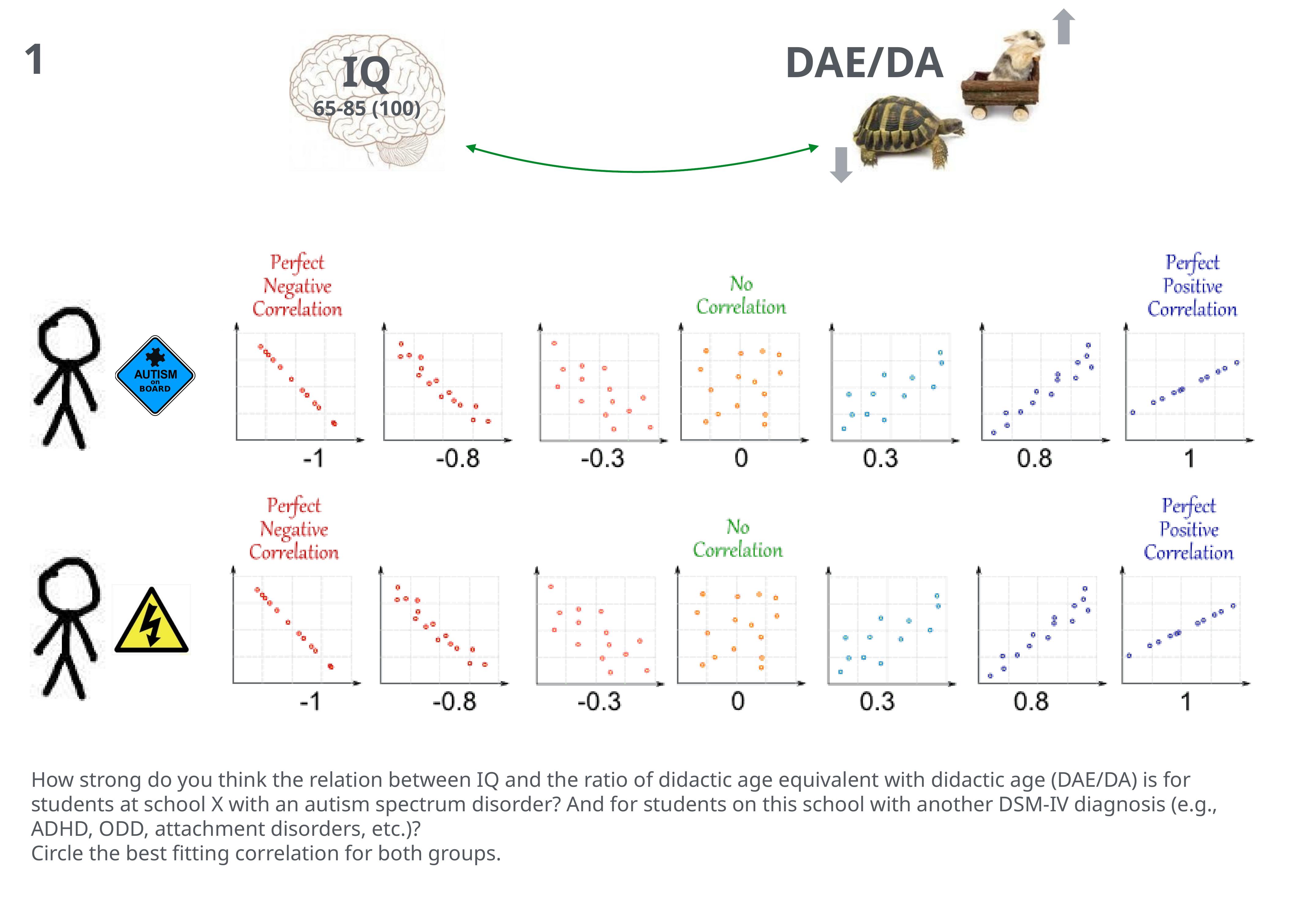

1
DAE/DA
IQ
65-85 (100)
How strong do you think the relation between IQ and the ratio of didactic age equivalent with didactic age (DAE/DA) is for students at school X with an autism spectrum disorder? And for students on this school with another DSM-IV diagnosis (e.g., ADHD, ODD, attachment disorders, etc.)?
Circle the best fitting correlation for both groups.

## Slide 7
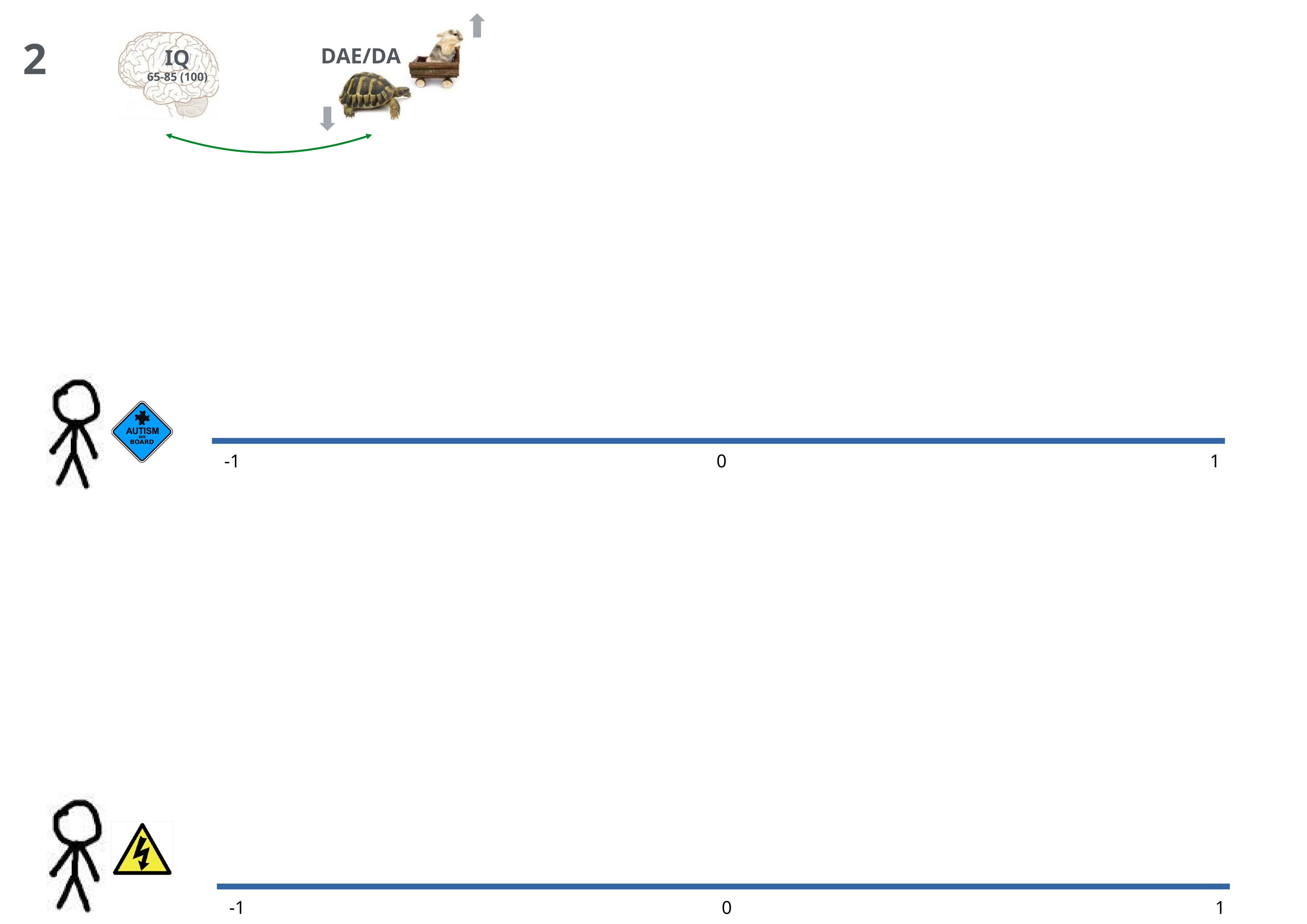

2
IQ
65-85 (100)
DAE/DA
-1
0
1
-1
0
1

## Slide 8
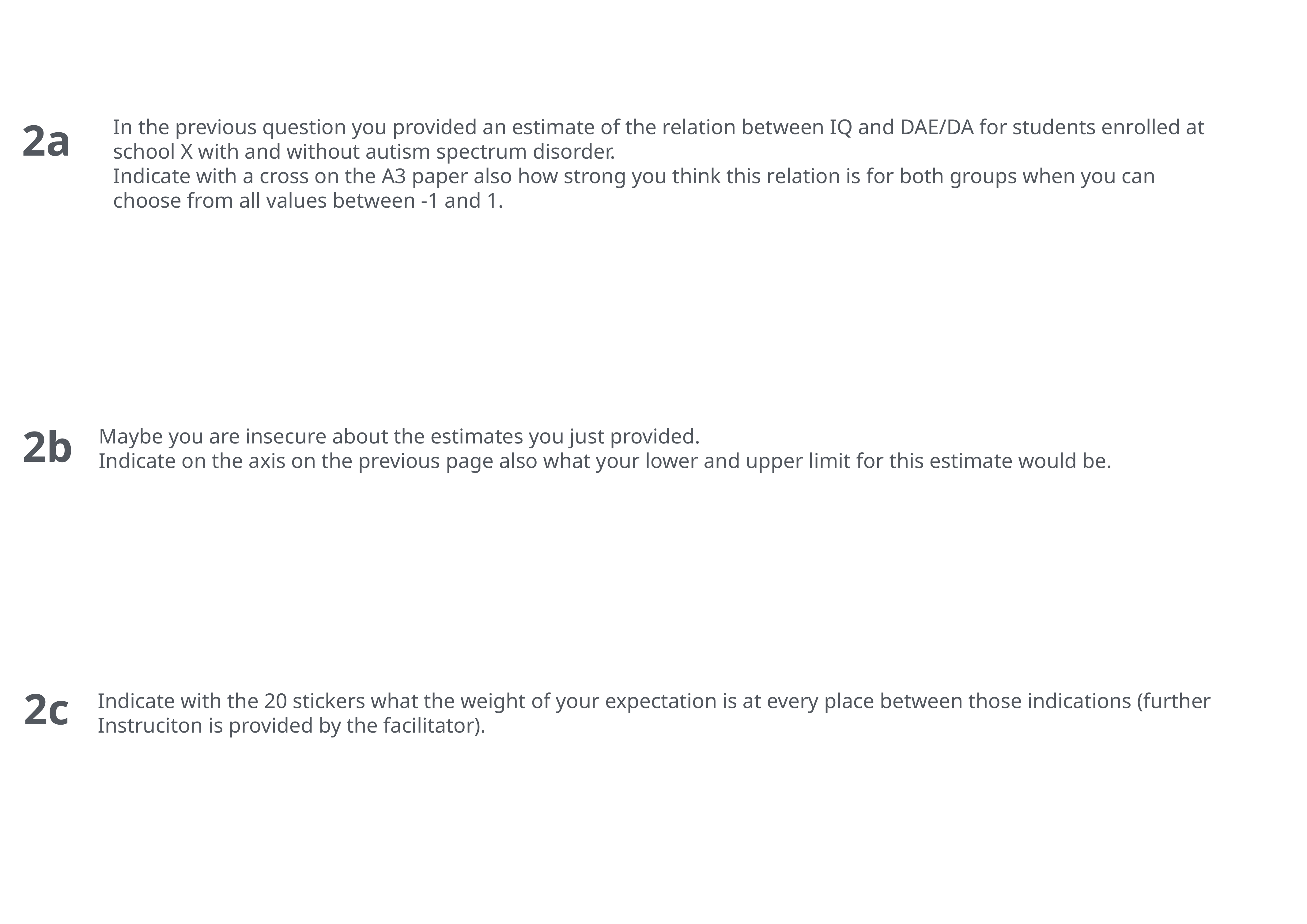

2a
In the previous question you provided an estimate of the relation between IQ and DAE/DA for students enrolled at school X with and without autism spectrum disorder.
Indicate with a cross on the A3 paper also how strong you think this relation is for both groups when you can choose from all values between -1 and 1.
2b
Maybe you are insecure about the estimates you just provided.
Indicate on the axis on the previous page also what your lower and upper limit for this estimate would be.
2c
Indicate with the 20 stickers what the weight of your expectation is at every place between those indications (further
Instruciton is provided by the facilitator).

## Slide 9
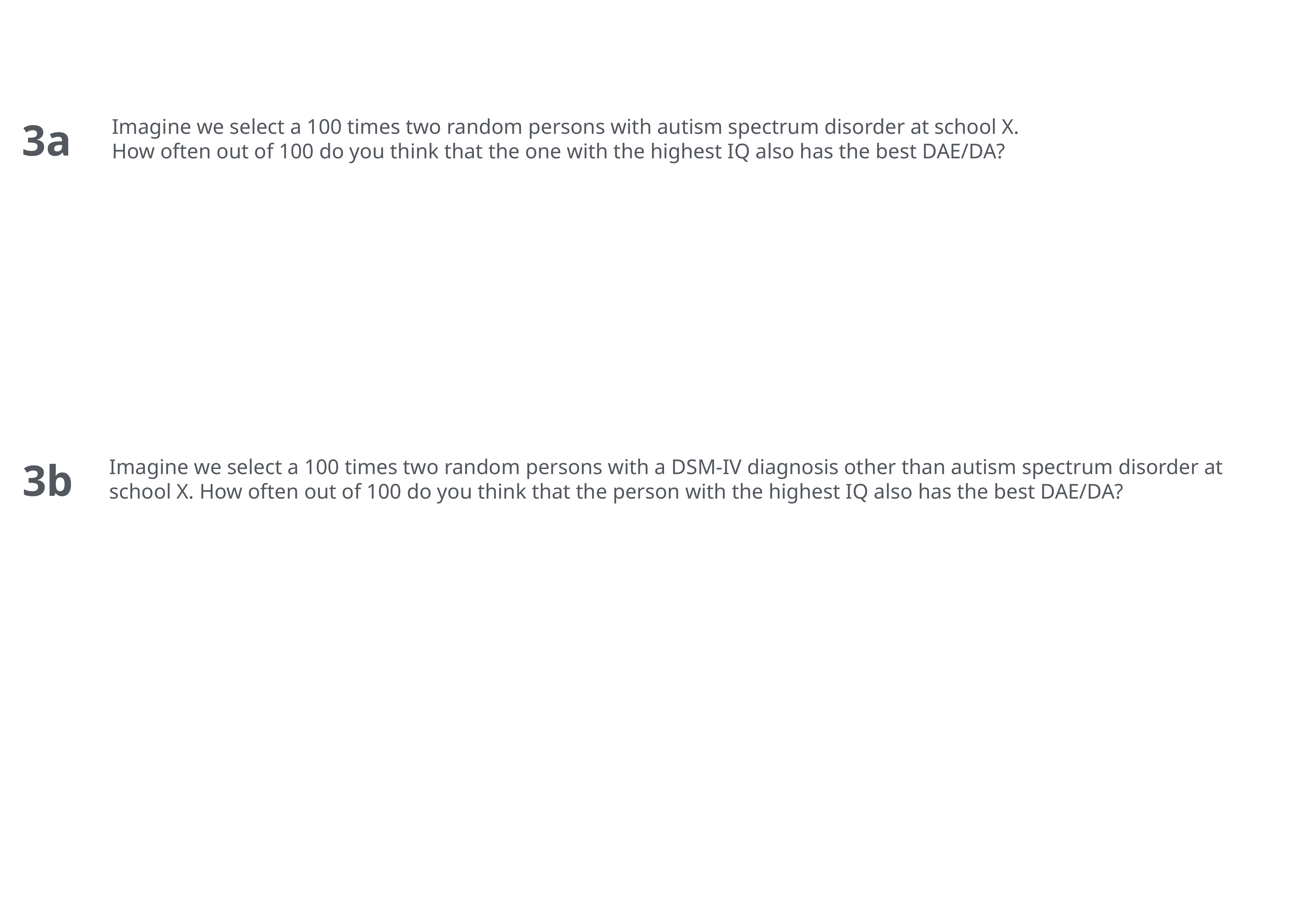

3a
Imagine we select a 100 times two random persons with autism spectrum disorder at school X.
How often out of 100 do you think that the one with the highest IQ also has the best DAE/DA?
3b
Imagine we select a 100 times two random persons with a DSM-IV diagnosis other than autism spectrum disorder at
school X. How often out of 100 do you think that the person with the highest IQ also has the best DAE/DA?

## Slide 10
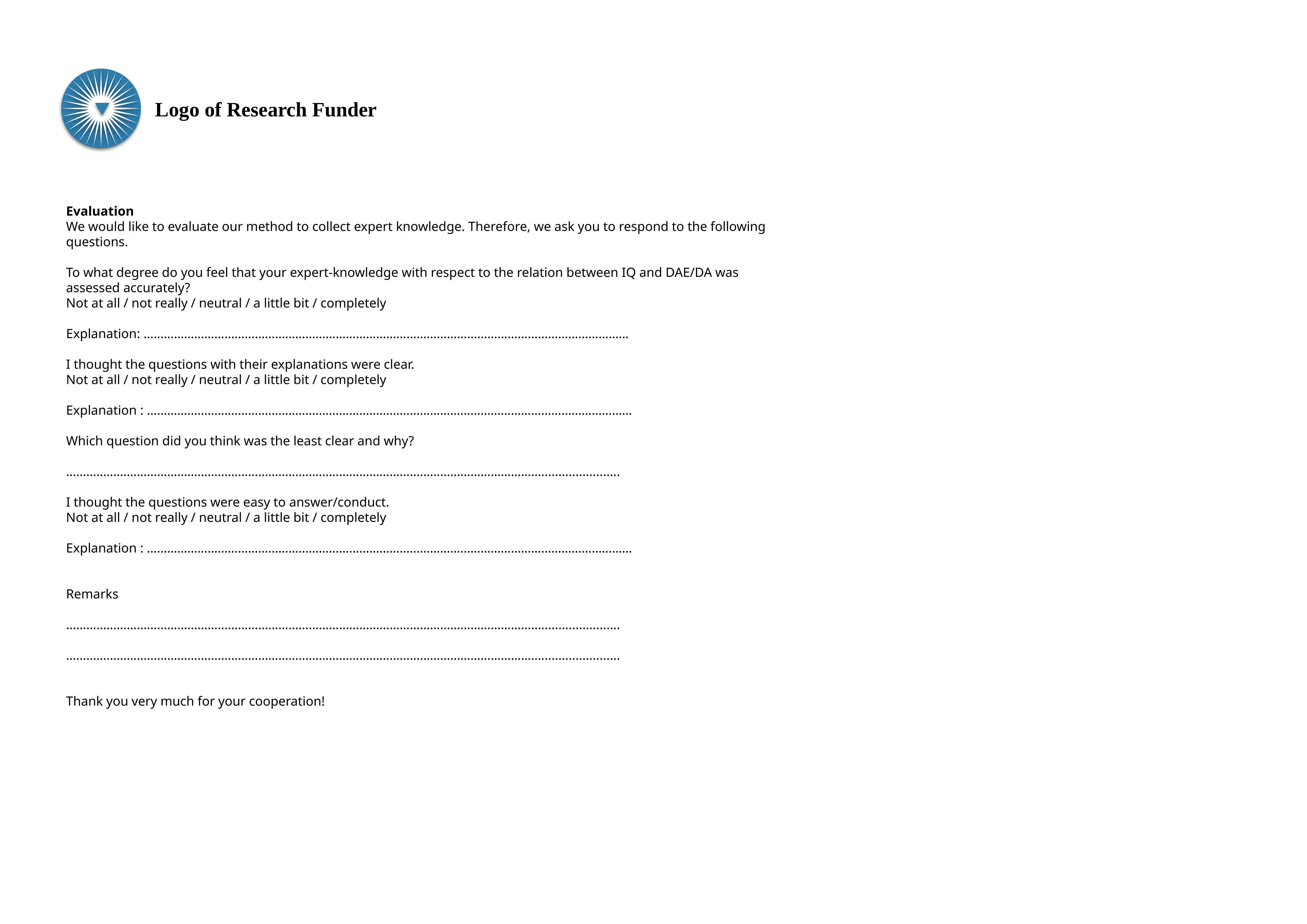

Logo of Research Funder
Evaluation
We would like to evaluate our method to collect expert knowledge. Therefore, we ask you to respond to the following questions.
To what degree do you feel that your expert-knowledge with respect to the relation between IQ and DAE/DA was assessed accurately?
Not at all / not really / neutral / a little bit / completely
Explanation: ………………………………………………………………………………………………………………………………
I thought the questions with their explanations were clear.
Not at all / not really / neutral / a little bit / completely
Explanation : ………………………………………………………………………………………………………………………………
Which question did you think was the least clear and why?
…………………………………………………………………………………………………………………………….......................
I thought the questions were easy to answer/conduct.
Not at all / not really / neutral / a little bit / completely
Explanation : ………………………………………………………………………………………………………………………………
Remarks
…………………………………………………………………………………………………………………………….......................
…………………………………………………………………………………………………………………………….......................
Thank you very much for your cooperation!
