# Supplementary material for: Application and Evaluation of an Expert Judgment Elicitation Procedure for Correlations
Source: Front Psychol. 2017 Jan 31;8:90. doi: 10.3389/fpsyg.2017.00090 (PMC5282462; doi:10.3389/fpsyg.2017.00090)
Supplement: Supplementary file 3 [file Part_IV_Bayesian_update.DOCX]

#####################################################################################

# In this script R-code is provided to conduct the analyses described in the paper

##IQdata ####

library(foreign)

library(dplyr)

library(mice)

library(mvtnorm)

library(ggplot2)

library(coda)

select <- dplyr::select #code to make dplyr's select work when MASS is in use

calc_age <- function(birthDate, refDate = Sys.Date()) {

require(lubridate)

period <- as.period(new_interval(birthDate, refDate),unit = "years")

period$year}

#make sure the data file is in the working directory folder

Data <- read.csv("Part III - Data.csv")

#save correlations from data in r.data

r.data<-NA

DataASD <- Data %>%

filter(ASD==1) %>%

select(IQ,DLEDL)

r.data[1] <- cor(DataASD)[1,2]

DatanoASD <- Data %>%

filter(ASD==0) %>%

select(IQ,DLEDL)

r.data[2] <- cor(DatanoASD)[1,2]

r.settings = length(r.data)

png("Data%01d.png")

plot(DataASD, xlim=c(50,100),ylim=c(0,1.0),bty="n",ylab="DAE/DA")

text(x=90,y=.10,labels=substitute(paste(italic('r'), " = .11 [-.52, .67]" )))

plot(DatanoASD, xlim=c(50,100),ylim=c(0,1.0),bty="n",ylab="DAE/DA")

text(x=90,y=.10,labels=substitute(paste(italic('r'), " = .32 [-.44, .81]" )))

dev.off()

#prior parameters

#rho1

#expert 1 lognormal, hyperparameters mu & sigma:

e1a.h1 <- -0.3543455; e1a.h2 <- 0.1163011

#expert 2 & 3 beta, hyperparameters

e2a.h1 <- 8.520659; e2a.h2 <- 7.167028

e3a.h1 <- 14.973041; e3a.h2 <- 7.181388

#expert 4 normal, hyperparameters mu & sigma:

e4a.h1 <- 0.70803613; e4a.h2 <- 0.08189341

#rho2

#expert 1-2 normal, hyperparameters mu & sigma:

e1b.h1 <- 0.4620081; e1b.h2 <- 0.12047800

e2b.h1 <- 0.2520595; e2b.h2 <- 0.04674401

#expert 3 beta, hyperparameters:

e3b.h1 <- 21.72679; e3b.h2 <- 34.25861

#expert 4 gamma, hyperparameters shape & scale:

e4b.h1 <- 32.76447; e4b.h2 <- 1/65.41338

lim = 1; nprior=500000; set.seed(2015)

library(Runuran)

#sample expert priors with weight 1/4 each

e1a <- urlnorm(nprior/4,e1a.h1,e1a.h2,ub=lim)

e2a <- urbeta(nprior/4,e2a.h1,e2a.h2,lb=-lim,ub=lim)

e3a <- urbeta(nprior/4,e3a.h1,e3a.h2,lb=-lim,ub=lim)

e4a <- urnorm(nprior/4,e4a.h1,e4a.h2,lb=-lim,ub=lim)

e1b <- urnorm(nprior/4,e1b.h1,e1b.h2,lb=-lim,ub=lim)

e2b <- urnorm(nprior/4,e2b.h1,e2b.h2,lb=-lim,ub=lim)

e3b <- urbeta(nprior/4,e3b.h1,e3b.h2,lb=-lim,ub=lim)

e4b <- urgamma(nprior/4,shape=e4b.h1,scale=e4b.h2,lb=-lim,ub=lim)

#combine expert priors

rho1 <- c(e1a,e2a,e3a,e4a)

rho2 <- c(e1b,e2b,e3b,e4b)

#normal priors on mu's

mu1_0.1 <- 75.0; mu1_0.2 <- 75.0

s1_0.1 <- 400.0; s1_0.2 <- 400.0

mu2_0.1 <- 0.75; mu2_0.2 <- 0.75

s2_0.1 <- 0.5; s2_0.2 <- 0.5

#sigma1[]-sigma2[] <- dgamma(shape,1/scale)

shape1 = 2.0; scale1 = 7.5

iscale1 =1/scale1

shape2 = 2.0; scale2 = 1/5.5

iscale2=1/scale2

#visualize, uninformative analysis ####

library(rjags)

n.iter = 50000; n.burnin=5000;

n.chains = 3

# analysis rho1 ####

n.iter = 50000; n.burnin=5000;

n.chains = 3

#expert 1 lognormal, hyperparameters mu & tau:

e1a.h1 <- -0.3543455; e1a.h2 <- 1/0.1163011^2

#expert 2 & 3 beta, hyperparameters

e2a.h1 <- 8.520659; e2a.h2 <- 7.167028

e3a.h1 <- 14.973041; e3a.h2 <- 7.181388

#expert 4 normal, hyperparameters mu & tau:

e4a.h1 <- 0.70803613; e4a.h2 <- 1/0.08189341^2

model_1a <- "

model {

for(i in 1:n) {

x[i,1:2] ~ dmnorm(mu2[], prec2[ , ])

}

# Constructing the covariance matrix and the corresponding precision matrix.

prec2[1:2,1:2] <- inverse(cov2[,])

cov2[1,1] <- sigma2[1] * sigma2[1]

cov2[1,2] <- sigma2[1] * sigma2[2] * rho1

cov2[2,1] <- sigma2[1] * sigma2[2] * rho1

cov2[2,2] <- sigma2[2] * sigma2[2]

# Priors

#normal priors on mu's

mu2[1] ~ dnorm(mu1_0.2,1/s1_0.2)T(45,145)

mu2[2] ~ dnorm(mu2_0.2,1/s2_0.2)T(0,1.5)

#gamma priors on sigma's

sigma2[1] ~ dgamma(shape1,iscale1)

sigma2[2] ~ dgamma(shape2,iscale2)

#prior for correlations group 1, expert 1

rho1 ~ dlnorm(e1a.h1,e1a.h2) T(-1,1)

}

"

model_2a <- "

model {

for(i in 1:n) {

x[i,1:2] ~ dmnorm(mu2[], prec2[ , ])

}

# Constructing the covariance matrix and the corresponding precision matrix.

prec2[1:2,1:2] <- inverse(cov2[,])

cov2[1,1] <- sigma2[1] * sigma2[1]

cov2[1,2] <- sigma2[1] * sigma2[2] * rho1

cov2[2,1] <- sigma2[1] * sigma2[2] * rho1

cov2[2,2] <- sigma2[2] * sigma2[2]

# Priors

#normal priors on mu's

mu2[1] ~ dnorm(mu1_0.2,1/s1_0.2)T(45,145)

mu2[2] ~ dnorm(mu2_0.2,1/s2_0.2)T(0,1.5)

#gamma priors on sigma's

sigma2[1] ~ dgamma(shape1,iscale1)

sigma2[2] ~ dgamma(shape2,iscale2)

#prior for correlations group 1, expert 2

rho1 ~ dbeta(e2a.h1,e2a.h2) T(-1,1)

}

"

model_3a <- "

model {

for(i in 1:n) {

x[i,1:2] ~ dmnorm(mu2[], prec2[ , ])

}

# Constructing the covariance matrix and the corresponding precision matrix.

prec2[1:2,1:2] <- inverse(cov2[,])

cov2[1,1] <- sigma2[1] * sigma2[1]

cov2[1,2] <- sigma2[1] * sigma2[2] * rho1

cov2[2,1] <- sigma2[1] * sigma2[2] * rho1

cov2[2,2] <- sigma2[2] * sigma2[2]

# Priors

#normal priors on mu's

mu2[1] ~ dnorm(mu1_0.2,1/s1_0.2)T(45,145)

mu2[2] ~ dnorm(mu2_0.2,1/s2_0.2)T(0,1.5)

#gamma priors on sigma's

sigma2[1] ~ dgamma(shape1,iscale1)

sigma2[2] ~ dgamma(shape2,iscale2)

#prior for correlations expert 3

rho1 ~ dbeta(e3a.h1,e3a.h2) T(-1,1)

}

"

model_4a <- "

model {

for(i in 1:n) {

x[i,1:2] ~ dmnorm(mu2[], prec2[ , ])

}

# Constructing the covariance matrix and the corresponding precision matrix.

prec2[1:2,1:2] <- inverse(cov2[,])

cov2[1,1] <- sigma2[1] * sigma2[1]

cov2[1,2] <- sigma2[1] * sigma2[2] * rho1

cov2[2,1] <- sigma2[1] * sigma2[2] * rho1

cov2[2,2] <- sigma2[2] * sigma2[2]

# Priors

#normal priors on mu's

mu2[1] ~ dnorm(mu1_0.2,1/s1_0.2)T(45,145)

mu2[2] ~ dnorm(mu2_0.2,1/s2_0.2)T(0,1.5)

#gamma priors on sigma's

sigma2[1] ~ dgamma(shape1,iscale1)

sigma2[2] ~ dgamma(shape2,iscale2)

#prior for correlations group 1 (a) and 2 (b) per expert

rho1 ~ dnorm(e4a.h1,e4a.h2) T(-1,1)

}

"

data_list = list(x = DataASD, n = nrow(DataASD),

mu1_0.2=mu1_0.2, mu2_0.2=mu2_0.2, s1_0.2=s1_0.2, s2_0.2 = s2_0.2,

e1a.h1 = e1a.h1, e2a.h1 = e2a.h1, e3a.h1 = e3a.h1, e4a.h1 = e4a.h1,

e1a.h2 = e1a.h2, e2a.h2 = e2a.h2, e3a.h2 = e3a.h2, e4a.h2 = e4a.h2,

shape1 = shape1, iscale1 =iscale1,shape2 = shape2, iscale2 =iscale2)

# Use classical estimates of the parameters as initial values

inits_list_a = list(rho1=cor(DataASD)[2],

mu2 = c(mean(DataASD[,1]),mean(DataASD[,2])),

sigma2 = c(sd(DataASD[,1]),sd(DataASD[,2])))

#run model ##########################################################################

par(mfrow = c(2, 2), mar = rep(2, 4))

jags_model <- jags.model(textConnection(model_1a), data = data_list, inits = inits_list_a,

n.adapt = 500, n.chains = n.chains, quiet = T)

update(jags_model, n.burnin)

mcmc_samples.1a <- coda.samples(jags_model, c("mu2", "rho1", "sigma2"),

n.iter = n.iter)

jags_model <- jags.model(textConnection(model_2a), data = data_list, inits = inits_list_a,

n.adapt = 500, n.chains = n.chains, quiet = T)

update(jags_model, n.burnin)

mcmc_samples.2a <- coda.samples(jags_model, c("mu2", "rho1", "sigma2"),

n.iter = n.iter)

jags_model <- jags.model(textConnection(model_3a), data = data_list, inits = inits_list_a,

n.adapt = 500, n.chains = n.chains, quiet = T)

update(jags_model, n.burnin)

mcmc_samples.3a <- coda.samples(jags_model, c("mu2", "rho1", "sigma2"),

n.iter = n.iter)

jags_model <- jags.model(textConnection(model_4a), data = data_list, inits = inits_list_a,

n.adapt = 500, n.chains = n.chains, quiet = T)

update(jags_model, n.burnin)

mcmc_samples.4a <- coda.samples(jags_model, c("mu2", "rho1", "sigma2"),

n.iter = n.iter)

png("ConvergenceChainA%01d.png")

par(mfrow = c(3, 2), mar = rep(2, 4))

plot(mcmc_samples.1a, auto.layout = FALSE,cex.main=1,cex.axis=0.8,mgp = c(2, 0.5, 0))

plot(mcmc_samples.2a, auto.layout = FALSE,cex.main=1,cex.axis=0.8,mgp = c(2, 0.5, 0))

plot(mcmc_samples.3a, auto.layout = FALSE,cex.main=1,cex.axis=0.8,mgp = c(2, 0.5, 0))

plot(mcmc_samples.4a, auto.layout = FALSE,cex.main=1,cex.axis=0.8,mgp = c(2, 0.5, 0))

dev.off()

#combine the posterior iterations for each chain for the correlation (i.e., [,3])

mcmc_samples.1a.r <- cbind(mcmc_samples.1a[[1]][,3],mcmc_samples.1a[[2]][,3],mcmc_samples.1a[[3]][,3])

mcmc_samples.2a.r <- cbind(mcmc_samples.2a[[1]][,3],mcmc_samples.2a[[2]][,3],mcmc_samples.2a[[3]][,3])

mcmc_samples.3a.r <- cbind(mcmc_samples.3a[[1]][,3],mcmc_samples.3a[[2]][,3],mcmc_samples.3a[[3]][,3])

mcmc_samples.4a.r <- cbind(mcmc_samples.4a[[1]][,3],mcmc_samples.4a[[2]][,3],mcmc_samples.4a[[3]][,3])

mcmc_samples.a.r <- array(c(mcmc_samples.1a.r,mcmc_samples.2a.r,mcmc_samples.3a.r,mcmc_samples.4a.r),

dim=c(n.iter,n.chains,4)) #rows=iterations, col=chains, d3=experts

#combine the posterior iterations for each chain for the se of iq (i.e., [,4])

mcmc_samples.1a.sIQ <- cbind(mcmc_samples.1a[[1]][,4],mcmc_samples.1a[[2]][,4],mcmc_samples.1a[[3]][,4])

mcmc_samples.2a.sIQ <- cbind(mcmc_samples.2a[[1]][,4],mcmc_samples.2a[[2]][,4],mcmc_samples.2a[[3]][,4])

mcmc_samples.3a.sIQ <- cbind(mcmc_samples.3a[[1]][,4],mcmc_samples.3a[[2]][,4],mcmc_samples.3a[[3]][,4])

mcmc_samples.4a.sIQ <- cbind(mcmc_samples.4a[[1]][,4],mcmc_samples.4a[[2]][,4],mcmc_samples.4a[[3]][,4])

mcmc_samples.a.sIQ <- array(c(mcmc_samples.1a.sIQ,mcmc_samples.2a.sIQ,mcmc_samples.3a.sIQ,mcmc_samples.4a.sIQ),

dim=c(n.iter,n.chains,4)) #rows=iterations, col=chains, d3=experts

#combine the posterior iterations for each chain for the se of dle/dl (i.e., [,5])

mcmc_samples.1a.sDL <- cbind(mcmc_samples.1a[[1]][,5],mcmc_samples.1a[[2]][,5],mcmc_samples.1a[[3]][,5])

mcmc_samples.2a.sDL <- cbind(mcmc_samples.2a[[1]][,5],mcmc_samples.2a[[2]][,5],mcmc_samples.2a[[3]][,5])

mcmc_samples.3a.sDL <- cbind(mcmc_samples.3a[[1]][,5],mcmc_samples.3a[[2]][,5],mcmc_samples.3a[[3]][,5])

mcmc_samples.4a.sDL <- cbind(mcmc_samples.4a[[1]][,5],mcmc_samples.4a[[2]][,5],mcmc_samples.4a[[3]][,5])

mcmc_samples.a.sDL <- array(c(mcmc_samples.1a.sDL,mcmc_samples.2a.sDL,mcmc_samples.3a.sDL,mcmc_samples.4a.sDL),

dim=c(n.iter,n.chains,4))

summary(mcmc_samples.a.sIQ)

summary(mcmc_samples.a.sDL)

# PSR convergence ####

PSR <- matrix(NA,nrow=n.iter,ncol=4)

theta.est.c <- matrix(NA,nrow=3,ncol=4); theta.est <- matrix(NA,nrow=1,ncol=4)

B <- matrix(NA,nrow=1,ncol=4); W <- matrix(NA,nrow=1,ncol=4)

for (p in 1:4){ #four posteriors

for (i in seq(from=1,to=n.iter-99,by=100)){ #every 100 iterations

theta.est.c[,p] <- colMeans(mcmc_samples.a.r[i:(i+99),,p]) #averages within chain over 100 it

theta.est[,p] <- mean(theta.est.c[,p]) #average over chains

B[,p] <- 1/(n.chains-1)*(sum((theta.est.c[,p]-theta.est[,p])^2)) #var between chains

W[,p] <- mean(colMeans((mcmc_samples.a.r[i:(i+99),,p]-theta.est.c[,p])^2)) #var within chains

PSR[i,p] <- sqrt((W[,p]+B[,p])/W[,p])

}}

PSR <- PSR[seq(from=1,to=n.iter-99,by=100),]

for (p in 1:4){print(sum(PSR[,p]<1.05)/length(PSR[,p])*100)

print(sum(PSR[,p]<1.01)/length(PSR[,p])*100)}

par(mfrow=c(1,1))

png("PosteriorA.png",width=480*1.25,height=280*1.25)

plot(density(mcmc_samples.a.r),lwd=3,xlab="",main="",xlim=c(-1,1),ylim=c(0,12),bty="n")

dev.off()

#prior, data, posterior plot a####

#relative profile likelihood function

rho.prof.like <- function(x,r,n){(((1-r^2)*(1-x^2))/(1-r*x)^2)^(n/2)}

png("PriorDataPosteriorA.png",width=480*1.25,height=280*1.25)

plot(density(mcmc_samples.a.r),lwd=3,xlab="",main="",xlim=c(-1,1),ylim=c(0,12),bty="n",col="#386cb0")

curve(rho.prof.like(x,r=r.data[1],n=dim(DataASD)[1]),col="#fb9a99",lwd=3,add=TRUE)

lines(density(rho1),lwd=2.5,col="#5ab4ac")

legend(-1,8,c("Prior","Likelihood","Posterior"),col=c("#5ab4ac","#fb9a99","#386cb0"),

lty=1,lwd=2.5,bty="n")

dev.off()

#table input A####

#prior

round(mean(e1a),2);round(mean(e2a),2);round(mean(e3a),2);round(mean(e4a),2)

round(HPDinterval(mcmc(e1a)),2);round(HPDinterval(mcmc(e2a)),2)

round(HPDinterval(mcmc(e3a)),2);round(HPDinterval(mcmc(e4a)),2)

#pooled prior

round(mean(rho1),2); round(HPDinterval(mcmc(rho1)),2)

#data

cor.test(DataASD[,1],DataASD[,2])[4]

round(cor.test(DataASD[,1],DataASD[,2])$conf.int,2)

#informative posterior

round(mean(mcmc_samples.1a.r),2)

round(HPDinterval(mcmc(as.vector(mcmc_samples.1a.r))),2)

round(mean(mcmc_samples.2a.r),2)

round(HPDinterval(mcmc(as.vector(mcmc_samples.2a.r))),2)

round(mean(mcmc_samples.3a.r),2)

round(HPDinterval(mcmc(as.vector(mcmc_samples.3a.r))),2)

round(mean(mcmc_samples.4a.r),2)

round(HPDinterval(mcmc(as.vector(mcmc_samples.4a.r))),2)

#pooled posterior

round(mean(mcmc_samples.a.r),2)

round(HPDinterval(mcmc(as.vector(mcmc_samples.a.r))),2)

#analysis rho2 ####

#expert 1-2 normal, hyperparameters mu & tau:

e1b.h1 <- 0.4620081; e1b.h2 <- 1/0.12047800^2

e2b.h1 <- 0.2520595; e2b.h2 <- 1/0.04674401^2

#expert 3 beta, hyperparameters:

e3b.h1 <- 21.72679; e3b.h2 <- 34.25861

#expert 4 gamma, hyperparameters shape & iscale:

e4b.h1 <- 32.76447; e4b.h2 <- 65.41338

model_1 <- "

model {

for(i in 1:n) {

x[i,1:2] ~ dmnorm(mu2[], prec2[ , ])

}

# Constructing the covariance matrix and the corresponding precision matrix.

prec2[1:2,1:2] <- inverse(cov2[,])

cov2[1,1] <- sigma2[1] * sigma2[1]

cov2[1,2] <- sigma2[1] * sigma2[2] * rho2

cov2[2,1] <- sigma2[1] * sigma2[2] * rho2

cov2[2,2] <- sigma2[2] * sigma2[2]

# Priors

#normal priors on mu's

mu2[1] ~ dnorm(mu1_0.2,1/s1_0.2)T(45,145)

mu2[2] ~ dnorm(mu2_0.2,1/s2_0.2)T(0,1.5)

#gamma priors on sigma's

sigma2[1] ~ dgamma(shape1,iscale1)

sigma2[2] ~ dgamma(shape2,iscale2)

#prior for correlations group 1, expert 1

rho2 ~ dnorm(e1b.h1,e1b.h2) T(-1,1)

}

"

model_2 <- "

model {

for(i in 1:n) {

x[i,1:2] ~ dmnorm(mu2[], prec2[ , ])

}

# Constructing the covariance matrix and the corresponding precision matrix.

prec2[1:2,1:2] <- inverse(cov2[,])

cov2[1,1] <- sigma2[1] * sigma2[1]

cov2[1,2] <- sigma2[1] * sigma2[2] * rho2

cov2[2,1] <- sigma2[1] * sigma2[2] * rho2

cov2[2,2] <- sigma2[2] * sigma2[2]

# Priors

#normal priors on mu's

mu2[1] ~ dnorm(mu1_0.2,1/s1_0.2)T(45,145)

mu2[2] ~ dnorm(mu2_0.2,1/s2_0.2)T(0,1.5)

#gamma priors on sigma's

sigma2[1] ~ dgamma(shape1,iscale1)

sigma2[2] ~ dgamma(shape2,iscale2)

#prior for correlations group 1, expert 2

rho2 ~ dnorm(e2b.h1,e2b.h2) T(-1,1)

}

"

model_3 <- "

model {

for(i in 1:n) {

x[i,1:2] ~ dmnorm(mu2[], prec2[ , ])

}

# Constructing the covariance matrix and the corresponding precision matrix.

prec2[1:2,1:2] <- inverse(cov2[,])

cov2[1,1] <- sigma2[1] * sigma2[1]

cov2[1,2] <- sigma2[1] * sigma2[2] * rho2

cov2[2,1] <- sigma2[1] * sigma2[2] * rho2

cov2[2,2] <- sigma2[2] * sigma2[2]

# Priors

#normal priors on mu's

mu2[1] ~ dnorm(mu1_0.2,1/s1_0.2)T(45,145)

mu2[2] ~ dnorm(mu2_0.2,1/s2_0.2)T(0,1.5)

#gamma priors on sigma's

sigma2[1] ~ dgamma(shape1,iscale1)

sigma2[2] ~ dgamma(shape2,iscale2)

#prior for correlations expert 3

rho2 ~ dbeta(e3b.h1,e3b.h2) T(-1,1)

}

"

model_4 <- "

model {

for(i in 1:n) {

x[i,1:2] ~ dmnorm(mu2[], prec2[ , ])

}

# Constructing the covariance matrix and the corresponding precision matrix.

prec2[1:2,1:2] <- inverse(cov2[,])

cov2[1,1] <- sigma2[1] * sigma2[1]

cov2[1,2] <- sigma2[1] * sigma2[2] * rho2

cov2[2,1] <- sigma2[1] * sigma2[2] * rho2

cov2[2,2] <- sigma2[2] * sigma2[2]

# Priors

#normal priors on mu's

mu2[1] ~ dnorm(mu1_0.2,1/s1_0.2)T(45,145)

mu2[2] ~ dnorm(mu2_0.2,1/s2_0.2)T(0,1.5)

#gamma priors on sigma's

sigma2[1] ~ dgamma(shape1,iscale1)

sigma2[2] ~ dgamma(shape2,iscale2)

#prior for correlations group 1 (a) and 2 (b) per expert

rho2 ~ dgamma(e4b.h1,e4b.h2) T(-1,1)

}

"

data_list = list(x = DatanoASD, n = nrow(DatanoASD),

mu1_0.2=mu1_0.2, mu2_0.2=mu2_0.2, s1_0.2=s1_0.2, s2_0.2 = s2_0.2,

e1b.h1 = e1b.h1, e2b.h1 = e2b.h1, e3b.h1 = e3b.h1, e4b.h1 = e4b.h1,

e1b.h2 = e1b.h2, e2b.h2 = e2b.h2, e3b.h2 = e3b.h2, e4b.h2 = e4b.h2,

shape1 = shape1, iscale1 =iscale1,shape2 = shape2, iscale2 =iscale2)

# Use classical estimates of the parameters as initial values

inits_list = list(rho2=cor(DatanoASD)[2],

mu2 = c(mean(DatanoASD[,1]),mean(DatanoASD[,2])),

sigma2 = c(sd(DatanoASD[,1]),sd(DatanoASD[,2])))

#run model ##########################################################################

jags_model <- jags.model(textConnection(model_1), data = data_list, inits = inits_list,

n.adapt = 500, n.chains = n.chains, quiet = T)

update(jags_model, n.burnin)

mcmc_samples.1 <- coda.samples(jags_model, c("mu2", "rho2", "sigma2"),

n.iter = n.iter)

par(mfrow = c(2, 2), mar = rep(2, 4))

jags_model <- jags.model(textConnection(model_2), data = data_list, inits = inits_list,

n.adapt = 500, n.chains = n.chains, quiet = T)

update(jags_model, n.burnin)

mcmc_samples.2 <- coda.samples(jags_model, c("mu2", "rho2", "sigma2"),

n.iter = n.iter)

jags_model <- jags.model(textConnection(model_3), data = data_list, inits = inits_list,

n.adapt = 500, n.chains = n.chains, quiet = T)

update(jags_model, n.burnin)

mcmc_samples.3 <- coda.samples(jags_model, c("mu2", "rho2", "sigma2"),

n.iter = n.iter)

jags_model <- jags.model(textConnection(model_4), data = data_list, inits = inits_list,

n.adapt = 500, n.chains = n.chains, quiet = T)

update(jags_model, n.burnin)

mcmc_samples.4 <- coda.samples(jags_model, c("mu2", "rho2", "sigma2"),

n.iter = n.iter)

png("ConvergenceChainB%01d.png")

par(mfrow = c(3, 2), mar = rep(2, 4))

plot(mcmc_samples.1, auto.layout = FALSE,cex.main=1,cex.axis=0.8,mgp = c(2, 0.5, 0))

plot(mcmc_samples.2, auto.layout = FALSE,cex.main=1,cex.axis=0.8,mgp = c(2, 0.5, 0))

plot(mcmc_samples.3, auto.layout = FALSE,cex.main=1,cex.axis=0.8,mgp = c(2, 0.5, 0))

plot(mcmc_samples.4, auto.layout = FALSE,cex.main=1,cex.axis=0.8,mgp = c(2, 0.5, 0))

dev.off()

#combine the posterior iterations for each chain for the correlation (i.e., [,3])

mcmc_samples.1.r <- cbind(mcmc_samples.1[[1]][,3],mcmc_samples.1[[2]][,3],mcmc_samples.1[[3]][,3])

mcmc_samples.2.r <- cbind(mcmc_samples.2[[1]][,3],mcmc_samples.2[[2]][,3],mcmc_samples.2[[3]][,3])

mcmc_samples.3.r <- cbind(mcmc_samples.3[[1]][,3],mcmc_samples.3[[2]][,3],mcmc_samples.3[[3]][,3])

mcmc_samples.4.r <- cbind(mcmc_samples.4[[1]][,3],mcmc_samples.4[[2]][,3],mcmc_samples.4[[3]][,3])

mcmc_samples.r <- array(c(mcmc_samples.1.r,mcmc_samples.2.r,mcmc_samples.3.r,mcmc_samples.4.r),

dim=c(n.iter,n.chains,4)) #rows=iterations, col=chains, d3=experts

#combine the posterior iterations for each chain for the se of iq (i.e., [,4])

mcmc_samples.1b.sIQ <- cbind(mcmc_samples.1[[1]][,4],mcmc_samples.1[[2]][,4],mcmc_samples.1[[3]][,4])

mcmc_samples.2b.sIQ <- cbind(mcmc_samples.2[[1]][,4],mcmc_samples.2[[2]][,4],mcmc_samples.2[[3]][,4])

mcmc_samples.3b.sIQ <- cbind(mcmc_samples.3[[1]][,4],mcmc_samples.3[[2]][,4],mcmc_samples.3[[3]][,4])

mcmc_samples.4b.sIQ <- cbind(mcmc_samples.4[[1]][,4],mcmc_samples.4[[2]][,4],mcmc_samples.4[[3]][,4])

mcmc_samples.b.sIQ <- array(c(mcmc_samples.1b.sIQ,mcmc_samples.2b.sIQ,mcmc_samples.3b.sIQ,mcmc_samples.4b.sIQ),

dim=c(n.iter,n.chains,4)) #rows=iterations, col=chains, d3=experts

#combine the posterior iterations for each chain for the se of dle/dl (i.e., [,5])

mcmc_samples.1b.sDL <- cbind(mcmc_samples.1[[1]][,5],mcmc_samples.1[[2]][,5],mcmc_samples.1[[3]][,5])

mcmc_samples.2b.sDL <- cbind(mcmc_samples.2[[1]][,5],mcmc_samples.2[[2]][,5],mcmc_samples.2[[3]][,5])

mcmc_samples.3b.sDL <- cbind(mcmc_samples.3[[1]][,5],mcmc_samples.3[[2]][,5],mcmc_samples.3[[3]][,5])

mcmc_samples.4b.sDL <- cbind(mcmc_samples.4[[1]][,5],mcmc_samples.4[[2]][,5],mcmc_samples.4[[3]][,5])

mcmc_samples.b.sDL <- array(c(mcmc_samples.1b.sDL,mcmc_samples.2b.sDL,mcmc_samples.3b.sDL,mcmc_samples.4b.sDL),

dim=c(n.iter,n.chains,4))

summary(mcmc_samples.a.sIQ)

summary(mcmc_samples.a.sDL)

summary(mcmc_samples.b.sIQ)

summary(mcmc_samples.b.sDL)

# PSR convergence ####

PSR <- matrix(NA,nrow=n.iter,ncol=4)

theta.est.c <- matrix(NA,nrow=3,ncol=4); theta.est <- matrix(NA,nrow=1,ncol=4)

B <- matrix(NA,nrow=1,ncol=4); W <- matrix(NA,nrow=1,ncol=4)

for (p in 1:4){ #four posteriors

for (i in seq(from=1,to=n.iter-99,by=100)){ #every 100 iterations

theta.est.c[,p] <- colMeans(mcmc_samples.r[i:(i+99),,p]) #averages within chain over 100 it

theta.est[,p] <- mean(theta.est.c[,p]) #average over chains

B[,p] <- 1/(n.chains-1)*(sum((theta.est.c[,p]-theta.est[,p])^2)) #var between chains

W[,p] <- mean(colMeans((mcmc_samples.r[i:(i+99),,p]-theta.est.c[,p])^2)) #var within chains

PSR[i,p] <- sqrt((W[,p]+B[,p])/W[,p])

}}

PSR <- PSR[seq(from=1,to=n.iter-99,by=100),]

for (p in 1:4){print(sum(PSR[,p]<1.05)/length(PSR[,p])*100)

print(sum(PSR[,p]<1.01)/length(PSR[,p])*100)}

png("PosteriorB.png",width=480*1.25,height=280*1.25)

plot(density(mcmc_samples.r),lwd=3,xlab="",main="",xlim=c(-1,1),ylim=c(0,12),bty="n")

dev.off()

#prior, data, posterior plot b####

rho.prof.like <- function(x,r,n){(((1-r^2)*(1-x^2))/(1-r*x)^2)^(n/2)}

png("PriorDataPosteriorB.png",width=480*1.25,height=280*1.25)

plot(density(mcmc_samples.r),lwd=3,xlab="",main="",xlim=c(-1,1),ylim=c(0,12),bty="n",col="#386cb0")

curve(rho.prof.like(x,r=r.data[2],n=dim(DatanoASD)[1]),col="#fb9a99",lwd=3,add=TRUE)

lines(density(rho2),lwd=2.5,col="#5ab4ac")

legend(-1,8,c("Prior","Likelihood","Posterior"),col=c("#5ab4ac","#fb9a99","#386cb0"),

lty=1,lwd=2.5,bty="n")

dev.off()

#table input B####

#prior

round(mean(e1b),2);round(mean(e2b),2);round(mean(e3b),2);round(mean(e4b),2)

round(HPDinterval(mcmc(e1b)),2);round(HPDinterval(mcmc(e2b)),2)

round(HPDinterval(mcmc(e3b)),2);round(HPDinterval(mcmc(e4b)),2)

round(mean(rho2),2)

round(HPDinterval(mcmc(rho2)),2)

#data

cor.test(DatanoASD[,1],DatanoASD[,2])[4]

round(cor.test(DatanoASD[,1],DatanoASD[,2])$conf.int,2)

#informative posterior

round(mean(mcmc_samples.1.r),2)

round(HPDinterval(mcmc(as.vector(mcmc_samples.1.r))),2)

round(mean(mcmc_samples.2.r),2)

round(HPDinterval(mcmc(as.vector(mcmc_samples.2.r))),2)

round(mean(mcmc_samples.3.r),2)

round(HPDinterval(mcmc(as.vector(mcmc_samples.3.r))),2)

round(mean(mcmc_samples.4.r),2)

round(HPDinterval(mcmc(as.vector(mcmc_samples.4.r))),2)

#pooled posterior

round(mean(mcmc_samples.r),2)

round(HPDinterval(mcmc(as.vector(mcmc_samples.r))),2)

#sensitivity analysis

#rerun the analysis with the follow priors for the sds:

#sigma1[]-sigma2[] <- dgamma(shape,1/scale)

shape1 = .01; scale1 = 1/.01

iscale1 =1/scale1

shape2 = .01; scale2 = 1/.01

iscale2=1/scale2

#replacing the following code:

#sigma1[]-sigma2[] <- dgamma(shape,1/scale)

#shape1 = 2.0; scale1 = 7.5

#iscale1 =1/scale1

#shape2 = 2.0; scale2 = 1/5.5

#iscale2=1/scale2
