# Supplementary material for: Application and Evaluation of an Expert Judgment Elicitation Procedure for Correlations
Source: Front Psychol. 2017 Jan 31;8:90. doi: 10.3389/fpsyg.2017.00090 (PMC5282462; doi:10.3389/fpsyg.2017.00090)
Supplement: Supplementary file 5 [file Part_I_Development.PDF]

Supplementary Material, Part I: Application and Evaluation of an Expert Judgment Elicitation Procedure for Correlations

Mariëlle Zondervan-Zwijnenburg, Wenneke van de Schoot-Hubeek, Kimberley Lek, Herbert Hoijtink, and Rens van de Schoot

*Correspondence:
Mariëlle Zondervan-Zwijnenburg
M.A.J.Zwijnenburg@uu.nl

1 DEVELOPMENT OF THE ELICITATION PROCEDURE

The final judgment elicitation procedure was developed from an initial version through recurring interviews with key informants, and a pilot study. The following paragraphs describe key features of the initial judgment elicitation procedure, and how the key informants and pilot study contributed to the final version. The initial version of the procedure was based on the literature of O'Hagan et al. (2006); Johnson et al. (2010a,b); Clemen et al. (2000). The initial version already consisted of seven phases, but different from the final version it included (1) two potential illustrations to support the education phase with respect to correlations (see Figure S1 and S2), and (2) two potential feedback questions after the trial roulette question. The first feedback question was the concordance probability question, which is included in the final procedure, and explained in Appendix 1. In the initial procedure, however, two versions of the concordance probability question were considered. The first version asked how often the student with the highest cognitive potential would have the best academic performance, and the second version asked how often the student with the best academic performance would have the highest cognitive potential. Both versions assess the same question, and should result in the same answer, but the two versions were included to determine which format was preferred. The second feedback question assessed explained variance r^2 from which the correlation r could easily be calculated by taking the square root. In this question, experts were asked to represent the overlap between the two variables of interest by overlapping two sticky notes on an A4 paper (see Figure S3). The degree of overlap between the two sticky notes would subsequently be calculated by the facilitator. The assignment was clarified by asking: "In other words, what percentage of educational performance is explained by cognitive potential for the population of interest?".

1.1 Key Informant Interviews

Colleagues at the department of methods and statistics at Utrecht University, students of the Utrecht University research master Methodology and Statistics for the Behavioral, Biomedical, and Social Sciences (M&S[1]), and the second author (WH), who is a school psychologist, were repeatedly involved in the development of the elicitation procedure by means of interviews.

[1] http://www.uu.nl/masters/en/methodology-and-statistics-behavioural

A major decision based on these interviews was the choice between the included concordance probability feedback question and the overlap feedback question. Although overlap and its representation with sticky notes seem intuitive, it appeared difficult to make participants truly understand the question, and answer it based on their ideas about the correlation between cognitive potential and educational performance. Hence, the overlap feedback question was discarded in favor of the concordance probability feedback question. Additionally, the interviews led to the addition of the text: “The value indicates the strength of the relation (not its slope!)” to the correlation figure, to avoid that people confuse the correlation coefficient with a regression coefficient. Furthermore, the interviews with WH led to terminology improvements in the elicitation (e.g., ‘DSM-IV diagnosis’ instead of psychopathology), and the specific instruction for the trial roulette question prohibiting vertical overlap of stickers.

1.2 Pilot Testing

A pilot test was conducted in a class of the Utrecht University research master Development and Socialization in Childhood and Adolescence[2] ($N = 21$). The goal of the pilot test was to evaluate the elicitation procedure in general, and to make choices between different (versions of) education methods and feedback questions. Hence, the students were encouraged to write their feedback down for each question.

In the pilot test, the facilitator (i.e., first author: MZ) introduced the research, motivated the students, and discussed the two pictures to explain the meaning of correlations by means of a PowerPoint presentation. Subsequently, the students answered the trial roulette question and the concordance probability question with a pencil, paper, and removable stickers. The pilot test with research master students led to several conclusions about, and adaptations in, the elicitation procedure.

First, the alternative picture (Correlation and Dependence n.d., Figure S1) to explain correlations was disregarded. Figure S1 lacked boxes around the different sub-images, and contained too much information according to the students. Also, the original picture from MathIsFun.com to explain correlations (Pierce 2014; Figure S2) had sub-images ordered from 1 to -1. The suggestion of the students to reverse the order was implemented.

Second, the trial roulette question was received well. Students understood the assignment and rated the question as the best question to elicit correlations. One student suggested a further improvement of the questions’ explanation, which was adopted.

Third, the concordance probability question was considered most difficult by the students, but not too difficult. The two versions of the concordance probability question confused students: 42.9 percent of them changed their answer when the variables for the question were swapped, while the order of the variables technically does not make a difference. Several students expressed their insecurity about this question, because they thought the question was the same, but since it was asked twice, they felt that could not be true. Consequently, the large amount of students that had inconsistent responses was regarded a result of the question being asked twice, instead of a result of the specific order of variables. One student explicitly indicated that asking how often the person with the highest cognitive potential also has the highest academic performance matched the main question better than the other way around. Hence, this question was included in the final version, while the swapped version was discarded. The student also suggested to explain that an answer of 50 in this question relates to a correlation of 0. This explanation was also present in Clemen et al. (2000). The objective of the question in the context of the elicitation procedure, however, is to make experts think about the correlation from a different perspective. Hence, it

[2] http://www.uu.nl/masters/en/development-and-socialisation-childhood

was perceived better to provide the question to the experts without further explanation of its relation to correlations.

The key informant interviews, and pilot study resulted in the final version of the procedure to elicit judgments about correlations (as discussed in Appendix 1).

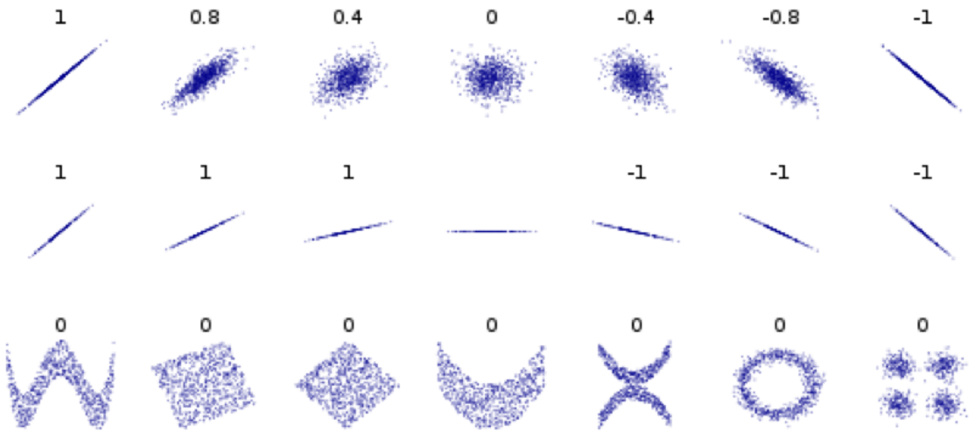

Figure S1. . Initial potential picture to explain correlations with from the English Wikipedia page on correlation and dependence (Correlation and dependence; n.d.).

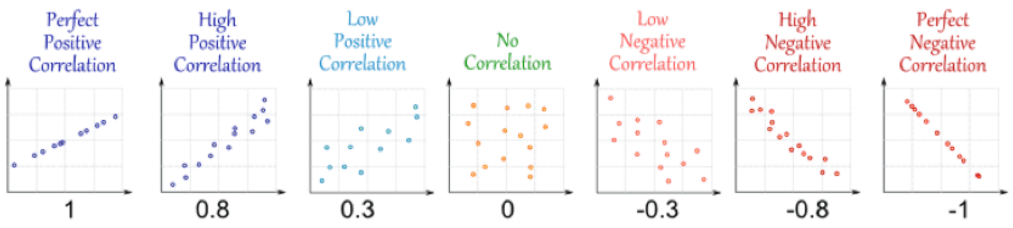

Figure S2. Initial potential picture to explain correlations with from MathIsFun.com (Pierce, 2014).

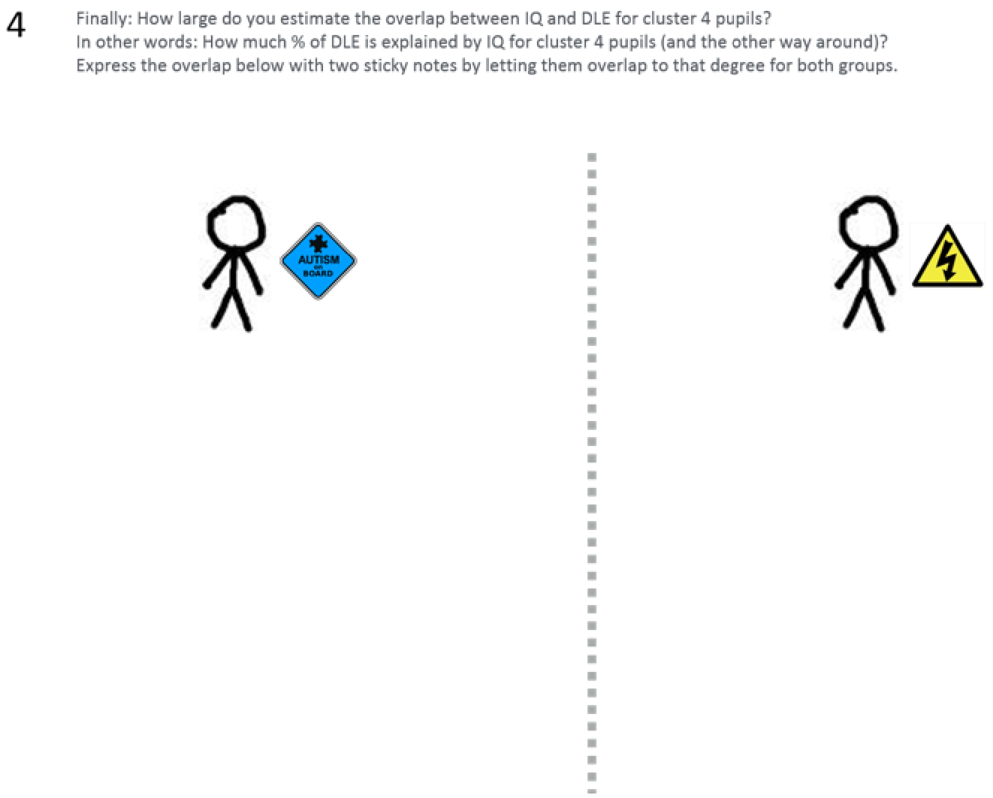

Figure S3. Initial potential feedback question 2: overlap.

REFERENCES

Clemen, R. T., Fischer, G. W., and Winkler, R. L. (2000). Assessing dependence: Some experimental results. *Management Science* 46, 1100–1115. doi:10.1287/mnsc.46.8.1100.12023

Correlation and Dependence (n.d.)

Johnson, S. R., Tomlinson, G. A., Hawker, G. A., Granton, J. T., and Feldman, B. M. (2010a). Methods to elicit beliefs for bayesian priors: a systematic review. *Journal of Clinical Epidemiology* 63, 355–369. doi:10.1016/j.jclinepi.2009.06.003

Johnson, S. R., Tomlinson, G. A., Hawker, G. A., Granton, J. T., Grosbein, H. A., and Feldman, B. M. (2010b). A valid and reliable belief elicitation method for Bayesian priors. *Journal of Clinical Epidemiology* 63, 370–383. doi:10.1016/j.jclinepi.2009.08.005

O'Hagan, A., Buck, C. E., Daneshkhah, A., Eiser, J. R., Garthwaite, P. H., Jenkinson, D. J., et al. (2006). *Uncertain judgements: eliciting experts' probabilities* (John Wiley & Sons). doi:10.1002/0470033312

Pierce, R. (2014). Correlation
