# Supplementary material for: Application and Evaluation of an Expert Judgment Elicitation Procedure for Correlations
Source: Front Psychol. 2017 Jan 31;8:90. doi: 10.3389/fpsyg.2017.00090 (PMC5282462; doi:10.3389/fpsyg.2017.00090)
Supplement: Supplementary file 6 [file Appendices.PDF]

Appendices: Application and Evaluation of an Expert Judgment Elicitation Procedure for Correlations

Mariëlle Zondervan-Zwijnenburg, Wenneke van de Schoot-Hubeek, Kimberley Lek, Herbert Hoijtink, and Rens van de Schoot
*Correspondence:
Mariëlle Zondervan-Zwijnenburg
M.A.J.Zwijnenburg@uu.nl

1 ELICITATION PROCEDURE INSTRUCTIONS

This appendix contains instructions for the elicitation procedure. The supporting presentation and questionnaire material is provided as online supplementary material (Part II).

1.1 Instructions

Terms & conditions: Ask for informed consent. Ask whether session can be recorded.

1. Motivation: Supported by slides, explain the goal of the elicitation, explain why the experts are important, explain that the process will help formalize their expertise into expectations about the correlations, explain that it is natural to be uncertain, explain that uncertainty can and should be expressed in answers as well, explain that questions can be asked at any time.
2. Clarification: Supported by slides, discuss central concepts like the research population, and the variables for which the correlation is elicited. Ask the experts: How would you describe <population of interest>? Do you ever run into <variable of interest>? Do you have an idea / could you explain what <variable of interest> stands for?
3. Education: Discuss and explain correlations with Figure **??**.
4. Instruction: Repeat that questions can be asked at any time, answers can be revised, and questions should be answered by experts at the same time so that questions can be discussed while answering them. Provide pencils with attached erasers.
5. Background Questions: Provide the questionnaire to the experts, and start the questionnaire. Wait until everyone has finished: ensure that the experts do not continue until everyone has finished the Background Questions.
6. Elicitation: Continue with the main questions, and ensure that questions are answered carefully, and simultaneously. Read question 1 of the elicitation questionnaire aloud and verify that the question is clear to everyone. Explain that it is an introductory question: later on, every value can be chosen instead of categories. Wait until everyone has finished.
   Read question 2a of the elicitation questionnaire aloud and verify that the question is clear to everyone. Wait until everyone has finished.

Read question 2b of the elicitation questionnaire aloud and verify that the question is clear to everyone. Wait until everyone has finished.

Read question 2c of the elicitation questionnaire aloud, and provide 20 stickers (for every distribution that is to be specified). Explain that they receive 20 stickers, each representing 5% that reflect probability: They should think for how probable it is that the correlation between <variable 1> and <variable 2> has of that specific value and attach stickers accordingly. Ask whether the experts are content with the distributions that they have specified. If not, they can adjust. Wait until everyone has finished.

Read question 3 of the elicitation questionnaire aloud and verify that the question is clear to everyone. Explain that question 3 is a new question, which they should think about independent of previous answers. Encourage the experts to forget everything they answered so far while responding to this question. Calculate the correlation matching the indicated concordance probability, and give this value as feedback to the expert. Ask the expert to re-evaluate the distribution in question 2 with this information. Ask whether the experts are satisfied with their answers, and continue when everyone is.

7. Evaluation: Provide experts with time and privacy to answer evaluation questions.

2 DIGITALIZING EXPERT JUDGMENT DISTRIBUTIONS TO CREATE HISTOGRAM PRIORS

In this appendix the digitalization of the expert judgment distributions is explained in detail. The result of the digitalization are histogram distributions. The histogram distributions can be used as priors in Bayesian analyses, but also serve as input to obtain parametric priors (see Appendix 3).

The digitalization of an expert's judgment distribution for the correlation consists of the following three steps:

Step 1: Divide the range of possible correlation values from -1 to $+1$ into $b = 1, ..., f$ intervals (i.e., bars) with an equal width.

Step 2: For $b = 1, ..., f$, set $p_b = 0$, where p_b denotes the probability mass assigned t a bar.

Step 3: Do for $l =, ..., k$,
If $b \in [\text{LL}_l, \text{UL}_l]$, then $p_b = p_b + \text{TP}_l/N_l$,
where l is a layer of stickers, k the number of layers, LL_l is the first bar within layer l, UL_l is the last bar within layer l, TP_l is the total probability in layer l (number of stickers in l times .05) and N_l the number of bars in the interval $[\text{LL}_l, \text{UL}_l]$.

Figure 2.1 illustrates Step 1: the application of bars to the expert judgment distribution. Here, $f = 80$, but only 40 bars are projected on the distribution itself to promote clarity, the 80 bars are displayed below the x-axis.

The annotated R-code matching the digitalization steps for the first distribution of expert 1 is:

```
#general settings
f = 80
b <- seq(1,f,by=1)
pb <- vector(length=f) #expert specific values k=5
#step 1
#step 2
```

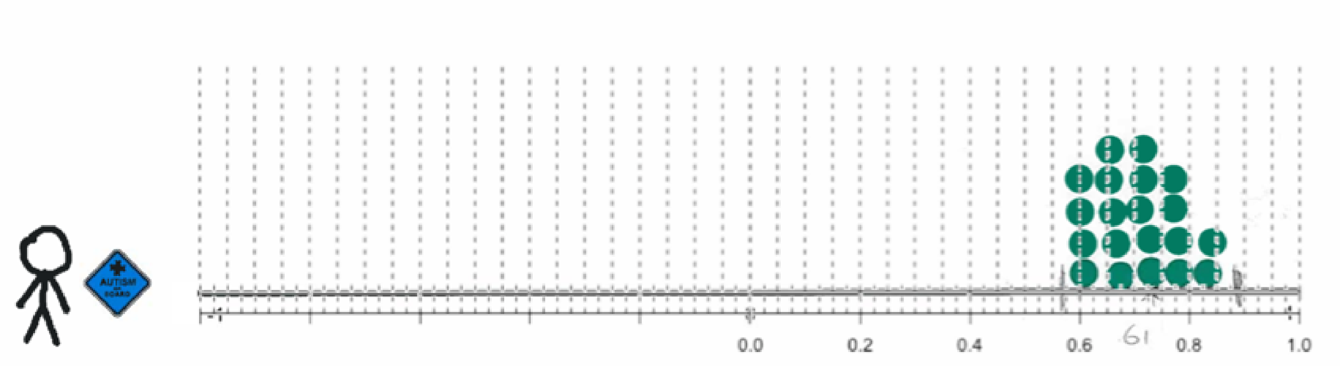

Figure 2.1. Distribution 1 for expert 1 with 80-bar grid (Step 1) applied.

```
LL <- c(64,64,64,64,66)
UL <- c(75,75,72,72,70)
TP <- c(0.25,0.25,0.20,0.20,0.10)
N <- c(12,12,9,9,5) #N <- 1+(UL-LL)
#step 3: for every bar, allocate probability from each associated layer for (
  for (l in 1:k){
    if (b[i] >= LL[l] & b[i] <= UL[l]){
    pb[i] = pb[i] + TP[l]/N[l]}
    }}
```

Histograms for digitalized trial roulette priors can be generated with the package LearnBayes (Albert, 2014) as follows:

```
midpt = seq(from=-1+2/f/2, to=1-2/f/2, by = 2/f)
p = seq(-1,1,length=2000)
plot(p,histprior(p,midpt,pb),type="l")
```

The result of this code is Figure 2.2.

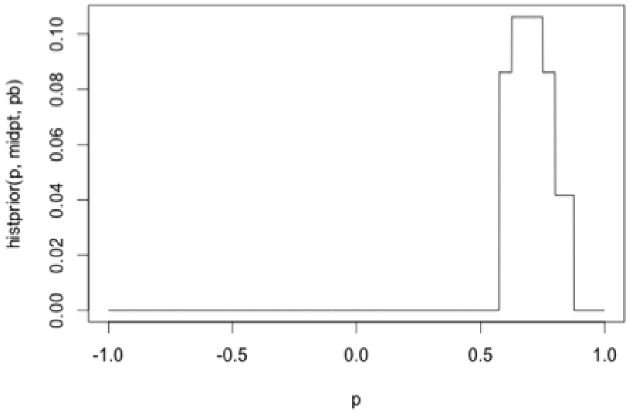

Figure 2.2. Correlation between cognitive potential and educational performance for youth with ASD according to expert 1.

3 DERIVE PARAMETRIC PRIORS FROM HISTOGRAM PRIORS

Prior distributions and hyperparameters matching the digitalized judgment distributions (Appendix 2) for the correlation can be found with the SHELF script. The function that should be used is:

```
elicit.group.values(N.experts = 4, method = "rp")
```

The function calls a window in which probability can be assigned to bars for the multiple experts. The probabilities of the each expert's vector pb were directly implemented. By making small adjustments to the SHELF script (nbins = 40 instead of 10), we were able to use 40 bars equally distributed over the interval (0,1), which was appropriate in our situation, because all experts indicated $p_b = 0$ for each bar in the interval (-1,0).
The resulting priors for the four experts and two populations were:

$$p_{1\text{ASD}}(\rho) = \ln N(\rho| - 0.354, 0.014) I_{\rho\in[0,1]} \tag{S1}$$

$$p_{2\text{ASD}}(\rho) = \text{Beta}(\rho|8.521, 7.167) \tag{S2}$$

$$p_{3\text{ASD}}(\rho) = \text{Beta}(\rho|14.973, 7.181) \tag{S3}$$

$$p_{4\text{ASD}}(\rho) = \ln N(\rho|0.708, 0.007) I_{\rho\in[0,1]} \tag{S4}$$

$$p_{1\text{noASD}}(\rho) = N(\rho|0.462, 0.015) I_{\rho\in[0,1]} \tag{S5}$$

$$p_{2\text{noASD}}(\rho) = N(\rho|0.252, 0.002) I_{\rho\in[0,1]} \tag{S6}$$

$$p_{3\text{noASD}}(\rho) = \text{Beta}(\rho|21.727, 34.259) \tag{S7}$$

$$p_{4\text{noASD}}(\rho) = \Gamma(\rho|32.224, 66.332) I_{\rho\in[0,1]}, \tag{S8}$$

where ASD refers to the population of youth with ASD, noASD refers to the population of youth with diagnoses other than ASD, N denotes a normal distribution with a mean and variance, Beta denotes a beta distribution with hyperparameters alpha and beta, and Γ denotes a gamma distribution with hyperparameters shape and rate.

4 CODE TO CONSTRUCT A POOL OF HISTOGRAM PRIORS

When the probabilities 'pb', as described in Appendix 2 are separately stored in vectors, the vectors can be simply added to construct the pooled histogram. For example, when the vectors are called pb1ASD, pb2ASD, pb3ASD, pb4ASD, pb1noASD, pb2noASD, pb3noASD, and pb4noASD, the pooled priors can be made with the following code:

```
pbASD <- pb1ASD + pb2ASD + pb3ASD + pb4ASD
pbnoASD <- pb1noASD + pb2noASD + pb3noASD + pbno4ASD
```

A histogram can then again be constructed with the package LearnBayes (Albert, 2014) as follows:

```
f = 80
midpt = seq(from=-1+2/f/2, to=1-2/f/2, by = 2/f)
p = seq(-1,1,length=2000)
plot(p,histprior(p,midpt,pbASD/sum(pbASD)),type="l")
```

In the last line of code where the plot is constructed, pbASD is divided by its sum (i.e., 400), to make the total integrate to 1 again.

5 CODE TO CONSTRUCT A POOL OF PARAMETRIC PRIORS

A figure of a parametric prior can be easily constructed with R-code specifying each density, its weight (e.g., 1 divided by the number of experts for equal priors), and subsequently adding the densities. With the parametric priors described in Appendix 3, the code to create a figure of the pooled distributions would be as follows:

```
curve(1/4*dlnorm(x,-0.3543455,0.1163011) +
1/4*dbeta(x,8.520659,7.167028) +
1/4*dbeta(x,14.973041,7.181388) +
1/4*dnorm(x,0.70803613,0.08189341), ylab="")
curve(1/4*dnorm(x,0.4620081,0.12047800) +
1/4*dnorm(x,0.2520595,0.04674401) +
1/4*dbeta(x,21.72679,34.25861) +
1/4*dgamma(x,32.76447,scale=1/65.41338), ylab="")
```

The pool of parametric distributions can be constructed by sampling from the separate parametric distributions and combining the resulting data. The sampling from truncated distributions can be done with the R-package Runuran (Leydold and Hörmann, 2015). The resulting pooled distribution, however, cannot be used as a prior directly. How a pooled parametric prior should be specified to be updated at once is software dependent. Some options are to write a sampler (Gill, 2014), write a module to add to existing software (Wabersich and Vandekerckhove, 2013), specify the (log)likelihood of the pooled parametric prior in Stan (Stan Development Team, 2014), or use the zeroes trick (Ntzoufras, 2009) in other software like OpenBUGS or Just Another Gibbs Sampler (JAGS; Plummer 2013).

6 BAYESIAN UPDATING

6.1 The Likelihood

The likelihood function of the model is given by:

$$p(x_1, y_1, ..., x_s, y_s|\mu_x, \mu_y, \sigma_x, \sigma_y, \rho) = \prod_{i=1}^{s} \frac{1}{2\pi\sigma_x\sigma_y\sqrt{1-\rho^2}} \exp\left[-\frac{1}{2(1-\rho^2)}\left[\left(\frac{x_i-\mu_x}{\sigma_x}\right)^2 + \left(\frac{y_i-\mu_y}{\sigma_y}\right)^2 - 2\rho\left(\frac{x_i-\mu_x}{\sigma_x}\right)\left(\frac{y_i-\mu_y}{\sigma_y}\right)\right]\right], \tag{S9}$$

where s denotes the number of subjects. In this bivariate normal distribution, the variance- covariance matrix is decomposed by means of the separation strategy (Barnard et al., 2000). This decomposition allows us to put a prior on ρ. Another advantage of this decomposition is that when $\rho \in [-1, 1]$, the variance-covariance matrix is always invertible.

6.2 Prior distributions

In the analysis for the population with ASD, the parametric expert priors were the following:

$$p_1(\rho) = \ln N(\rho| - 0.354, 0.014)I_{\rho\in[0,1]} \tag{S10}$$
$$p_2(\rho) = \text{Beta}(\rho|8.521, 7.167) \tag{S11}$$
$$p_3(\rho) = \text{Beta}(\rho|14.973, 7.181) \tag{S12}$$
$$p_4(\rho) = \ln N(\rho|0.708, 0.007)I_{\rho\in[0,1]} \tag{S13}$$

In the analysis for the population with diagnoses other than ASD, the parametric expert priors were:

$$p_1(\rho) = N(\rho|0.462, 0.015)I_{\rho\in[0,1]} \tag{S14}$$
$$p_2(\rho) = N(\rho|0.252, 0.002)I_{\rho\in[0,1]} \tag{S15}$$
$$p_3(\rho) = \text{Beta}(\rho|21.727, 34.259) \tag{S16}$$
$$p_4(\rho) = \Gamma(\rho|32.224, 66.332)I_{\rho\in[0,1]} \tag{S17}$$

In the equations, N denotes a normal distribution with a mean and variance, Beta denotes a beta distribution with hyperparameters alpha and beta, and Γ denotes a gamma distribution with hyperparameters shape and rate.

In addition to the prior distributions for the correlation, a joint prior for the nuisance parameters $(\mu_x, \mu_y, \sigma_x, \sigma_y)$ was specified:

$$\begin{aligned} p(\mu_x, \mu_y, \sigma_x, \sigma_y) = \\ N(\mu_x|75.0, 400, 0)I(\mu_x \in [45, 145]))\times \\ N(\mu_y|0.75, 0.50)I(\mu_y \in [0.0, 1.5]))\times \\ \Gamma(\sigma_x|2.0, \frac{1}{7.5}) \times \Gamma(\sigma_y|2.0, 5.5) \end{aligned} \tag{S18}$$

Justifications of the hyperparameters can be found in the main text, section **??**.

6.3 Posterior distribution

The posterior distribution is proportional to the prior times the likelihood of the data. The equation demonstrates this with a pooled expert prior.

$$\begin{aligned} p(\mu_x, \mu_y, \sigma_x, \sigma_y, \rho|x_1, y_1, ..., x_s, y_s) \propto \\ p(\mu_x, \mu_y, \sigma_x, \sigma_y) \times \sum_{e=1}^{4} \left(\frac{1}{4}p_e(\rho)\right) \times p(x_1, y_1, ..., x_s, y_s|\mu_x, \mu_y, \sigma_x, \sigma_y, \rho). \end{aligned} \tag{S19}$$

The summation symbol can be moved, since it only sums over elements with subscript e, giving:

$$p(\mu_x, \mu_y, \sigma_x, \sigma_y, \rho | x_1, y_1, ..., x_s, y_s) \propto \sum_{e=1}^{4} \Big(p(\mu_x, \mu_y, \sigma_x, \sigma_y) \times \frac{1}{4} p_e(\rho) \times p(x_1, y_1, ..., x_s, y_s | \mu_x, \mu_y, \sigma_x, \sigma_y, \rho) \Big). \quad \text{(S20)}$$

These equations demonstrate that updating a pooled prior with the likelihood of the data is equal to pooling the posteriors of four analysis in which only one element of the pooled distribution was updated with the likelihood of the data.
The posterior was thus obtained for each expert separately. From each posterior, parameters could be sampled by means of an iterative procedure, which could then be combined according to the linear pooling principle, resulting in a posterior distribution.

6.4 Analysis

All Bayesian analyses were conducted in JAGS (Plummer, 2013) via the package rjags (Plummer, 2015) in R (R Core Team, 2015). In JAGS, a Gibbs sampler is used to approximate the posterior. The number of chains in each analysis was 3. Each of the chains consisted of 5,000 burn-in iterations, and 50,000 post burn-in iterations. JAGS' automatic random number generators, and seed values were adopted. As starting values for the chains, the maximum likelihood estimates were provided. Annotated R-code is provided as online supplementary material (Part IV).

Convergence of the analyses was assessed by inspecting the trace plots, and evaluating the potential scale reduction (PSR; Gelman and Rubin 1992). For the population with ASD, the convergence plots looked satisfactory for all posterior distributions. In addition, the PSR for the correlation parameters was calculated for every 100 iterations. For each expert, the PSR was lower than 1.05 in more than 97.8% of the evaluations. For more than 56.0% of the evaluations, the PSR was lower than 1.01. For the population with diagnoses other than ASD, the convergence plots looked satisfactory for all posterior distributions. In addition, the PSR for the correlation parameters was calculated for every 100 iterations. For each expert, the PSR was lower than 1.05 in more than 99.8% of the evaluations. For more than 65.4% of the evaluations, the PSR was lower than 1.01.

REFERENCES

Albert, J. (2014). *LearnBayes: Functions for Learning Bayesian Inference*. R package version 2.15

Barnard, J., McCulloch, R., and Meng, X.-L. (2000). Modeling covariance matrices in terms of standard deviations and correlations, with application to shrinkage. *Statistica Sinica* 10, 1281–1312

Gelman, A. and Rubin, D. B. (1992). Inference from iterative simulation using multiple sequences. *Statistical Science* 7, 457–472. doi:10.1214/ss/1177011136

Gill, J. (2014). *Bayesian methods: A social and behavioral sciences approach*, vol. 20 (Boca Raton, FL: CRC press)

Leydold, J. and Hörmann, W. (2015). *Runuran: R Interface to the UNU.RAN Random Variate Generators*. R package version 0.23.0

Ntzoufras (2009). *Models for Positive Continuous Data, Count Data, and Other GLM-Based Extensions* (Hoboken, NJ: John Wiley & Sons). 275–304. doi:10.1002/9780470434567.ch8

Plummer, M. (2013). *JAGS Version 3.4.0 user manual computing [Computer software manual]*

Plummer, M. (2015). *rjags: Bayesian Graphical Models using MCMC*. R package version 3-15

R Core Team (2015). *R: A Language and Environment for Statistical Computing*. R Foundation for Statistical Computing, Vienna, Austria

Stan Development Team (2014). *Stan: A C++ library for probability and sampling (Version 2.11.0) [Software]*

Wabersich, D. and Vandekerckhove, J. (2013). Extending JAGS: A tutorial on adding custom distributions to JAGS (with a diffusion model example). *Behavioral Resarch Methods* 46, 15–28. doi:10.3758/s13428-013-0369-3
