# Supplementary material for: Application and Evaluation of an Expert Judgment Elicitation Procedure for Correlations
Source: Front Psychol. 2017 Jan 31;8:90. doi: 10.3389/fpsyg.2017.00090 (PMC5282462; doi:10.3389/fpsyg.2017.00090)
Supplement: Supplementary file 7 [file Part_III_validityandreliability.docx]

Supplementary Material: Data and R-code validity and reliability

In the following section, relevant data for the evaluation of validity and reliability is provided in an annotated R data frame. The trial roulette distributions are displayed elsewhere. Responses to open questions are not presented in the dataset to prevent that the individual experts can be identified. Subsequently, the R-code to evaluate the responses in terms of validity and reliability is provided.

asd.tau=c(71,70,75,80) #concordance probability*100 Autism Spectrum Disorder group

dsm.tau=c(58,60,60,70) #concordance probability*100 other DSM diagnoses

asd.tau.r=c(80,NA,75,NA)#concordance probability*100 ASD retest

dsm.tau.r=c(30,NA,40,NA)#concordance probability*100 no ASD retest

prior.data <- data.frame(asd1=c(0.8,0.8,0.8,0.8),dsm1=c(0.3,0.3,0.3,0.3), #Q1

pe.asd = c(.725,.525,.675,.725), #point estimate Q2 ASD

pe.dsm = c(.475,.200,.375,.500), #point estimate Q2 no ASD

asd.tau=c(71,70,75,80), #conc. prob. ASD

asd.rho=sin(1/2*(2*pi*asd.tau/100-pi)), #tau Q3 ASD

dsm.tau=c(58,60,60,70), #conc. prob. No ASD

dsm.rho=sin(1/2*(2*pi*dsm.tau/100-pi)), #tau Q3 no ASD

valid=c(4,2,4,4), #evaluation validity

clear=c(NA,4,5,5), #evaluation clearity

least=c(NA,1,2,NA), #evaluation, which question least clear

easy=c(4,4,4,5), #evaluation, ease

#retest scores

asd1.r=c(0.8,0.8,0.8,NA),dsm1.r=c(0.3,0.3,0.3,NA), #Q1

pe.asd.r = c(0.725,0.275,0.600,NA), #point estimate Q2 ASD

pe.dsm.r = c(0.200,0.275,0.350,NA), #point estimate Q2 no ASD

asd.tau.r=c(80,75,75,NA),

asd.rho.r=sin(1/2*(2*pi*asd.tau.r/100-pi)), #tau Q3 ASD

dsm.tau.r=c(30,60,40,NA),

dsm.rho.r=sin(1/2*(2*pi*dsm.tau.r/100-pi)) #tau Q3 no ASD

)

prior.data$valid; prior.data$clear; prior.data$easy

face_valid <- mean(prior.data$valid)

feasibility <- (mean(prior.data$clear,na.rm=TRUE)+mean(prior.data$easy,na.rm=TRUE))/2

conc_valid.ASD <- cor(prior.data$pe.asd,prior.data$asd.tau)

conc_valid.noASD <- cor(prior.data$pe.dsm,prior.data$dsm.tau)

conc_valid <- mean(c(conc_valid.ASD,conc_valid.noASD))

library(psych)

r.con(cor(prior.data$pe.dsm,prior.data$dsm.tau),n=4,p=.95)

r.con(cor(prior.data$pe.asd,prior.data$asd.tau),n=4,p=.95)

attach(prior.data)

mean(abs(pe.asd-asd.rho)) #mean absolute difference between point estimate and concordance prob. rho

mean(abs(pe.dsm-dsm.rho))

mean(c(abs(pe.asd-asd.rho),abs(pe.dsm-dsm.rho)))

validity <- round(matrix(c(face_valid,feasibility,conc_valid.ASD,conc_valid.noASD),nrow=4),2)

rownames(validity) <- c("face validity","feasibility","concordance validity ASD","concordance validity no ASD")

colnames(validity) <- "result"

validity

ICC(matrix(c(prior.data$pe.asd[1:3],prior.data$pe.asd.r[1:3]),nrow=2,byrow=TRUE))$results[5,2]

ICC(matrix(c(prior.data$pe.asd[1:3],prior.data$pe.asd.r[1:3]),nrow=2,byrow=TRUE))

ICC(matrix(c(prior.data$pe.dsm[1:3],prior.data$pe.dsm.r[1:3]),nrow=2,byrow=TRUE))$results[5,2]

ICC(matrix(c(prior.data$pe.dsm[1:3],prior.data$pe.dsm.r[1:3]),nrow=2,byrow=TRUE))

References

R Core Team. (2014). *R: A language and environment for statistical computing* [Computer software manual]. Vienna, Austria. Retrieved from <http://www.R-project.org>

Revelle, W. (2016) psych: Procedures for Personality and Psychological Research, Northwestern University, Evanston, Illinois, USA, https://CRAN.R-project.org/package=psych Version = 1.6.9.
